# Steroidomics for the Prevention, Assessment, and Management of Cancers: A Systematic Review and Functional Analysis

**DOI:** 10.3390/metabo9100199

**Published:** 2019-09-21

**Authors:** Nguyen Hoang Anh, Nguyen Phuoc Long, Sun Jo Kim, Jung Eun Min, Sang Jun Yoon, Hyung Min Kim, Eugine Yang, Eun Sook Hwang, Jeong Hill Park, Soon-Sun Hong, Sung Won Kwon

**Affiliations:** 1 College of Pharmacy, Seoul National University, Seoul 08826, Korea; 2018-23140@snu.ac.kr (N.H.A.); phuoclong@snu.ac.kr (N.P.L.); danielkim27@snu.ac.kr (S.J.K.); mje0107@snu.ac.kr (J.E.M.); supercanboy@snu.ac.kr (S.J.Y.); snuhmkim04@snu.ac.kr (H.M.K.); hillpark@snu.ac.kr (J.H.P.); 2 College of Pharmacy, Ewha Womans University, Seoul 03760, Korea; ginayang95@gmail.com (E.Y.); eshwang@ewha.ac.kr (E.S.H.); 3 Department of Biomedical Sciences, College of Medicine, Inha University, Incheon 22212, Korea; hongs@inha.ac.kr

**Keywords:** steroidomics, cancer, biomarker, diagnosis, prognosis, systematic review, functional analysis

## Abstract

Steroidomics, an analytical technique for steroid biomarker mining, has received much attention in recent years. This systematic review and functional analysis, following the PRISMA statement, aims to provide a comprehensive review and an appraisal of the developments and fundamental issues in steroid high-throughput analysis, with a focus on cancer research. We also discuss potential pitfalls and proposed recommendations for steroidomics-based clinical research. Forty-five studies met our inclusion criteria, with a focus on 12 types of cancer. Most studies focused on cancer risk prediction, followed by diagnosis, prognosis, and therapy monitoring. Prostate cancer was the most frequently studied cancer. Estradiol, dehydroepiandrosterone, and cortisol were mostly reported and altered in at least four types of cancer. Estrogen and estrogen metabolites were highly reported to associate with women-related cancers. Pathway enrichment analysis revealed that steroidogenesis; androgen and estrogen metabolism; and androstenedione metabolism were significantly altered in cancers. Our findings indicated that estradiol, dehydroepiandrosterone, cortisol, and estrogen metabolites, among others, could be considered oncosteroids. Despite noble achievements, significant shortcomings among the investigated studies were small sample sizes, cross-sectional designs, potential confounding factors, and problematic statistical approaches. More efforts are required to establish standardized procedures regarding study design, analytical procedures, and statistical inference.

## 1. Introduction

Metabolites are low-molecular-weight compounds, present in biological matrices as systemic outcomes of various metabolic cascades [1]. In other words, metabolites reflect the chemical regulations and transformations that occur within living organisms [2,3]. Metabolomics, the last member of the “omics” family, aims to identify and quantify metabolites such as amino acids, carbohydrates, and steroids existing in biological systems [4]. Currently, untargeted metabolomics and targeted metabolomics are the two main approaches of metabolic phenotyping research [5]. The untargeted approach aims to identify various metabolites, while the targeted approach focuses on a smaller number of metabolites under a specific condition or a metabolic pathway of interest [6]. A large body of evidence has suggested that metabolic profiling could be a beneficial approach for the assessment and management of human diseases [7]. More importantly, the flexibility and profound applicability of metabolomics, together with other omics, may assist with the development of translational and precision medicine [8,9,10].

Lipidomics is a sub-field of metabolomics that specifically targets lipids. It has many unique characteristics regarding the technical aspect [11]. Thus, lipids are usually analyzed separately from other more hydrophilic metabolites. Lipidomics has a wide range of applications, especially in the field of biomedical research [12]. Indeed, it could be implemented as a part of a mechanistic study or be applied for clinical biomarker discovery [13,14]. Among characterized metabolites, steroids belong to a family of molecules that play an essential role in cell structure formation and signal transduction [15]. In living organisms, steroids are mainly synthesized in the gonads, placenta, and adrenal cortex [6]. A disturbance in steroid homeostasis usually results in various disorders [16,17]. Recently, the roles of steroids in cancer have gained much attention, especially in endocrine and reproductive cancers. Indeed, there has been intensive research focusing on the dysregulations of steroids in adrenal cancer, breast cancer, and prostate cancer, to name a few [18,19,20]. Abnormal levels of steroid could be quantitatively measured to assist in the diagnosis and management of malignancies in clinical practice. For instance, the difference in steroid levels in biofluids and tissues between malignant and non-malignant conditions could be utilized to develop novel biomarkers for cancer risk prediction, diagnosis, and management [21,22,23].

Although the importance of steroid profiling has been demonstrated in cancer screening, diagnosis, prognosis, and management, comprehensive review and appraisal of the methodology are yet to be undertaken [24,25]. Additionally, possible pitfalls of analytical aspects in high-throughput steroid profiling have not been thoroughly described. These issues may hamper the translation of discovered biomarkers into clinical practice. Thus, this study was set out to systematically analyze and examine the latest research on the clinical functions of steroidomics in cancer. Moreover, current technical limitations, suboptimal methodological approaches, and the role of data analysis in steroid characterization were thoroughly reviewed.

## 2. Results

### 2.1. Synthesis of Literature and Search Strategies

**Figure 1:**
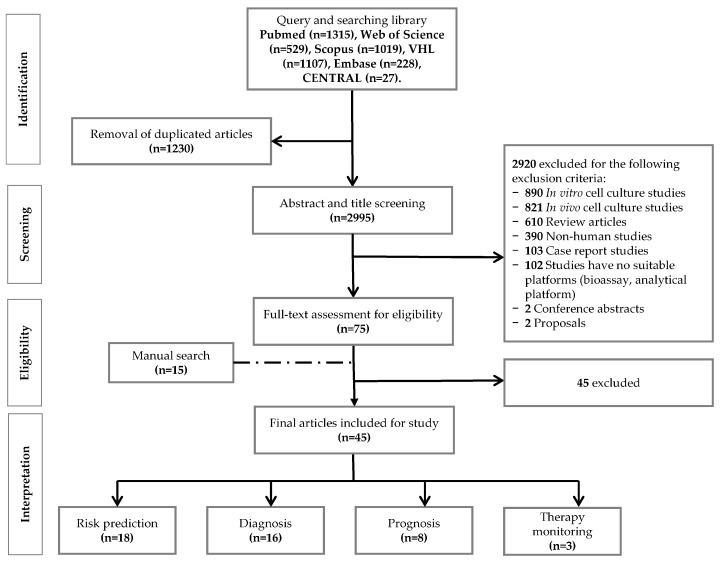
The flow diagram for the screening and the selection of suitable papers.

After the literature search, we retrieved 1315, 1019, 228, 529, 1107, and 27 articles from PubMed, Scopus, Embase, Web of Science Core Collection, Virtual Health Library (VHL), and Cochrane Controlled Register of Trials (CENTRAL), respectively. Subsequently, 1230 duplicate articles were removed, leaving 2995 references, which were screened for their title and abstract. After independent screening by at least two authors, 2920 papers were excluded in this step. In detail, 890, 821, 610, 390, 103, 102, two, and two papers were excluded for the following reasons: in vitro study, in vivo study, review article, non-human study, case report, no suitable platform, conference abstract, and proposal, respectively. Finally, the full-text of 75 eligible articles was carefully perused. While evaluating the articles, we found 15 additional papers by manual search. Finally, only 45 papers, 30 from the systematic search and 15 from the manual search, were suitable for data extraction. An overview of our study design and workflow of the record screening and assessment is given in Figure 1.

### 2.2. Characteristics of the Included Studies

Table 1 shows an overview of the population characteristics of the 45 included studies. Prostate cancer (PC) and adrenal cancer (AC) were the two most investigated diseases related to steroids, followed by breast cancer (BC) and endometrial cancer (EC). The roles of steroids were also explored in other types of cancer, such as ovarian cancer, esophageal and gastric cancer (E/GC), and liver cancer (LC). Pathological confirmation was primarily performed as the reference diagnostic method to confirm stages of cancer in 22 studies. Sample sizes in the cancer groups ranged from three to 1298 cases, while sample sizes of the control groups ranged from seven to 1524 cases, with one additional study recruiting 1776 patients in total. Among these studies, 33 studies had healthy individuals in the control group. It is worth noting that 18 studies utilized one or a mixture of non-cancerous conditions as control groups, such as a benign mass. In terms of study purpose, risk prediction (*n* = 18) and diagnosis (*n* = 16) were the primary outcomes among the studies. For cancer risk prediction, the association between estrogens and estrogen metabolites and cancer risk was the most reported. Regarding diagnosis purpose, adrenal cancer-related studies were the most predominant compared to other malignancies. Although there were many papers (*n* = 16) reporting diagnostic aspects of cancer, 13 of these studies either lacked information on stages of cancer or included late-stage (stage III-IV) cancers in the cohorts. Moreover, only approximately half of the included studies (*n* = 22) reported the follow-up period in their study. Figure S1 shows a descriptive summary of the included studies.

### 2.3. Sample Preparation and Analytical Procedures of the Included Studies

**Figure 2:**
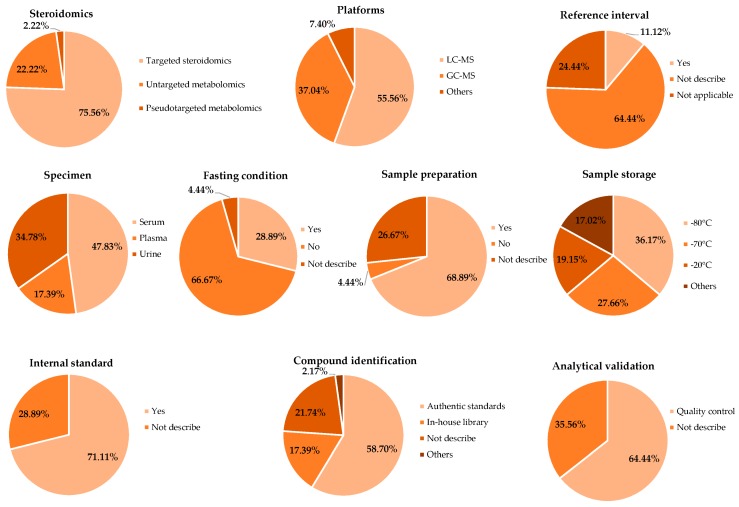
Study design and metabolomics approach of the included studies.

Figure 2 and Supplementary Table S1 summarize the sample characteristics and analytical procedure characteristics. Among 45 included studies there are 34 studies conducting a targeted steroid approach and the other studies (*n* = 11) reported steroid metabolites as part of the metabolite profiling results. Regarding the sample type, serum (*n* = 22), urine (*n* = 16), and plasma (*n* = 8) were the three types of biospecimen used in studies concerning steroid biomarkers. One study was conducted using both urine and serum samples. After collection, samples were commonly stored at −80 °C (*n* = 17) and −70 °C (*n* = 13), and about one-fifth of the studies stored samples at –20 °C. The liquid chromatography-mass spectrometry (LC-MS, *n* = 30) and gas chromatography-mass spectrometry (GC-MS, *n* = 20) were the most employed analytical platforms. Notably, eight studies employed both platforms; among them, three studies used targeted steroidomics, while the remaining five were untargeted metabolomics. Additionally, only four studies used other methods (open column, liquid chromatography with ultraviolet detection, liquid chromatography with diode array detection, and gas chromatography with flame ionization detector) for investigating the metabolites. The use of internal standard was a common practice and was observed in 32 studies, while 29 studies included quality control samples for the analytical validation. Authentic standards (*n* = 27) were the most frequently used method for steroid identification.

### 2.4. Steroids and the Prevention, Assessment, and Management of Cancer Patients

Most of the included studies in our analysis focused on steroid analysis to evaluate the associated metabolic processes. For instance, alterations in estrogen and androgen metabolism were associated with endocrine-related cancers, including prostate, breast, endometrial, and ovarian cancer [38,41,43,47,51]. Furthermore, esophagus and gastric cancer were also reported to have associations with commonly reported steroids such as dehydroepiandrosterone (DHEA), testosterone, and estradiol [39,40]. In a significant number of risk prediction studies, samples were withdrawn from a defined cohort [39,42,44,49]. Additionally, 10 included studies suggested the association between estrogen metabolism and cancer risk development.

Regarding the diagnostic aspect, most of the studies explored the alterations between the cancer patients and other groups, usually healthy controls. Of these, adrenocortical carcinoma (ACC) was mainly reported and usually distinguished from adrenocortical adenoma (ACA). Few studies reported steroid panels to classify cancer status and other conditions. For instance, Schweitzer and colleagues reported a group of steroid compounds predicting ACC, ACA, and healthy group with the high area under the curve (AUC) [26]. Since ACC is a rare disease, most ACC-related studies were conducted with small sample sizes compared to the other studies. It should, however, be noted that a considerable number of this type of research did not present follow-up reports, an important characteristic of diagnostic studies. It is also worth noting that there were two studies that characterized steroids on four types of cancer and suggested a panel of four steroids, which helped differentiate healthy and cancer samples with a sensitivity of 0.81 and a specificity of 0.74 [33] and another with a sensitivity of 1.00 and a specificity of 0.89 [23]. Primarily, the steroid level was suggested to have an essential role in the prognosis and therapy monitoring of cancers, such as prostate, breast, and liver cancer. However, it seemed that, except for cancer risk prediction and diagnosis, steroid profiling had not been well-studied in prognosis and therapy monitoring. Hence, there was no steroid panel suggested for these purposes. Most of the studies primarily monitored steroids associated with increased survival time or therapeutic efficacy. For instance, unconjugated estradiol was correlated with breast cancer and lower estriol levels correlated with higher recurrence rate and lower overall survival in EC patients [50,54]. The detailed results of each study are shown in Table S2. Table 2 summarizes the potential steroid biomarkers in cancer, which show a significant alteration in cancer compared to other groups.

### 2.5. Assessment of Reporting Methodology Quality

Thirty-nine out of 45 studies reported at least eight of the 16 quality assessment items. The six remaining studies reported at least six quality assessment items. Among these items, sample information, sample preparation, and reference standards were the most commonly reported, while 28 studies mentioned the inclusion and exclusion criteria in their studies. The detailed information is presented in Table S3.

### 2.6. Steroid Profiling Pathway Analysis and Network Analysis 

**Figure 3:**
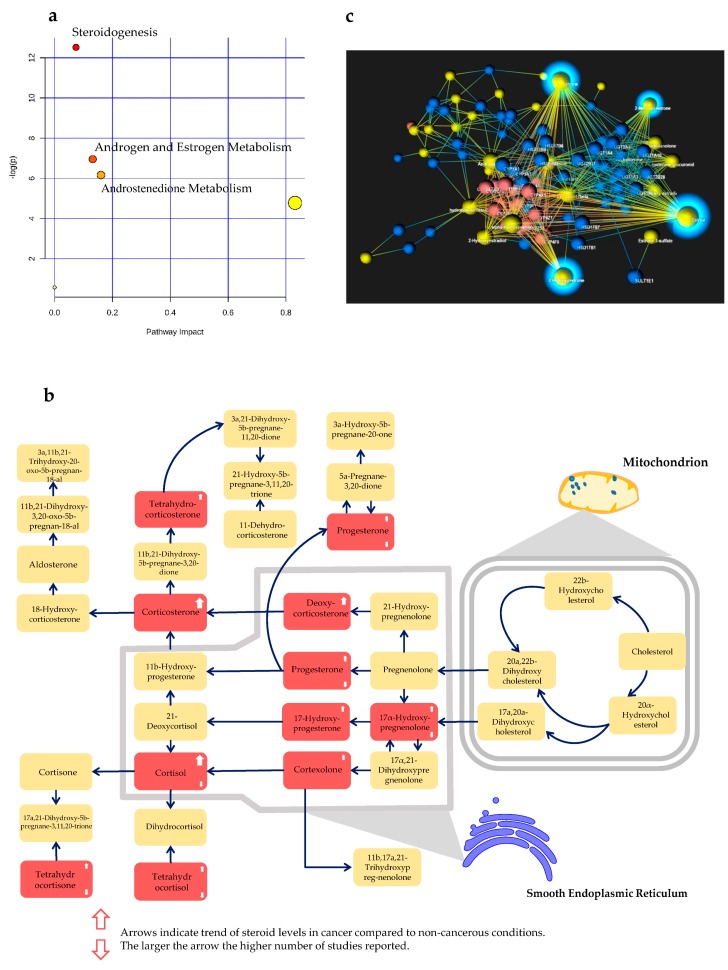
Associated biological processes of the steroid biomarkers reported in at least two studies. (**a**) Three significantly enriched pathway of the included steroids, (**b**) steroidogenesis pathway visualization and the altered steroids, the red boxes refer to the potentially altered steroids in the included studies, and (**c**) the network visualization of the steroids reported in at least two studies. Sparkling nodes indicate the central molecules in our network visualization.

We recorded 105 steroids throughout the included studies and 66 out of 105 steroids had the HMDB ID. Among 105 steroids, 55 steroids were reported as significantly dysregulated in cancers in at least two studies. Estradiol, DHEA, and cortisol were the three most reported steroids in 15, 11, and 10 studies, respectively, and displayed alterations across various types of cancer, such as AC, PC, and LC. Moreover, 15 steroids displayed remarkably altered levels in four different types of cancer. A full list of the steroid biomarkers is shown in Table S4. From 55 steroids, we extracted the official steroid nomenclature using Human Metabolome Database version 4.0. Eventually, only 43 steroids with available HMDB ID were included in the pathway enrichment and network analysis. As a result, steroid compounds were listed in three significant pathways including Steroidogenesis, Androgen and Estrogen metabolism, and Androstenedione metabolism, with false discovery rates (FDR) of 0.00036, 0.047, and 0.07, respectively. More details about the pathway visualization and pathway characteristics can be found in Figure 3a,b, Figure S2, and Table S5. In the network analysis using Kyoto Encyclopedia of Genes and Genomes (KEGG) pathway mapping, steroid hormone biosynthesis was the most significant pathway, followed by well-known pathways including Metabolism of xenobiotics by cytochrome P450 and Drug metabolism-cytochrome P450 (data not shown). Network visualization is shown in Figure 3c.

## 3. Discussion

Metabolomics, an informatively rich platform, has emerged as an essential approach to discover novel biomarkers for the prevention, assessment, and management of cancers [69]. The ability to use metabolites as a non-invasive, promising tool for differentiating between cancerous and non-malignant conditions has been proven in previous systematic assessments [70,71,72]. Nevertheless, steroid characterization for clinical utility, in particular, remains a challenging area of metabolomics due to the existence of confounding factors and the requirement of strict protocols for proper sample treatment [73,74,75]. In the present work, we explored the accumulated evidence of the role of steroidomics in the prevention, assessment, and management of cancers. Our comprehensive evaluation showed that various steroid species are significantly associated with common cancers, particularly BC, EC, PC, and E/GC. Researchers have made attempts to overcome potential issues and achieved remarkable results in this sphere. However, some aspects remained inconsistent and required further improvement. Thus far, most of the included studies have focused on seeking potential steroids that showed a significant level of changes between two different conditions and had been biased in the risk prediction and diagnostic domain. Nevertheless, no study investigated a robust panel of steroid utilized for cancer risk assessment. Regarding diagnosis, researchers have recently conducted steroid profiling in order to discover steroid diagnostic biomarkers, which could aid in differentiating different diseases or between cancer patients and healthy people. For example, Kerkhofs et al. and Arlt et al. conducted a mass spectrometric steroid analysis and suggested a steroid panel to distinguish ACC and ACA with outstanding performances [20,34]. In addition, Dai et al. discovered four potential steroids (cortisol, androstanediol, allo-tetrahydrocortisol, and epitestosterone), which were predictive of liver cirrhosis and early hepatocellular carcinoma with an AUC of 0.97 [31]. Similarly, a previous study showed that a biomarker panel demonstrated superior performance in cancer diagnosis and prognosis than a single biomarker [71]. In the current work, we proposed a list of commonly reported steroids that are associated with human cancer. These commonly reported steroids and their associated pathways could be utilized to establish an oncosteroid panel to improve prevention, assessment, and management of cancers.

Crucial information, such as age, gender, and follow-up, have not been well-described. It is notable that steroids are reported to demonstrate strong associations with these factors [73,76]. Age is identified as a significant confounder, with considerable variability of plasma steroids resulting from wide age ranges. For example, DHEA, one of the most reported steroids among the included studies, has a robust negative correlation with age. Therefore, the lack of control for age, gender, and hormone use history could significantly affect the result and lead to misinterpretation [75,77]. As mentioned earlier, steroid levels are gender and age-dependent and highly changeable due to differences in body mass index (BMI), sampling protocols, and history of hormone consumption, just to name a few [75,76]. Therefore, these classic confounding factors should be recognized and well-controlled. Data stratification during sampling and data analysis based on gender, age range, and underlying diseases should be considered. A standardized procedure in terms of time, fasting, and type of biofluid containers could also minimize the variability of steroid levels. Moreover, follow-up should be considered in the study design whenever possible, especially in the diagnostic investigation. Samples should be stored at temperatures less than or equal to −20 °C, preferably at −80 °C for long-term storage [75]. Additionally, long-term, prospective follow-up populations, which may provide more useful information, have been generally lacking [78,79]. The included studies mostly focused on cancer patients at late stages, but they may not be suitable for finding diagnostic biomarkers [80]. Finally, small sample size is also a common limitation, evidenced as only 20 studies had more than 100 samples in each group.

Qualitative and quantitative analyses of steroids demand proper and strict sample pretreatment processes [81]. Specifically, steroids can exist in conjugated and unconjugated forms in the human body. This leads to difficulties in setting up an efficient method for detecting steroidal compounds. Additionally, steroid levels greatly vary from serum, plasma, to urine [82]. Among commonly used biofluid samples, urine might be considered as the most non-invasive and intact sample compared to serum and plasma. In addition, urine profiling has been acknowledged as a crucial way to explore the disturbance of steroid synthesis [6]. However, high salt concentration might challenge the mass spectrometry assay when conducting urine profiling. Therefore, a pre-analytical step for separating the steroid from salt should be added before conducting the data acquisition. For example, the employment of solid-phase extraction can reduce the salt and concentrate the steroid [83]. In the case of serum and plasma profiling, the steroid level was strongly associated with diet and nutritional supply [75]. Therefore, fasting should be recommended when collecting the blood sample, where possible. Likewise, estrogens and testosterone were positively associated with BMI in postmenopausal women, with older postmenopausal women showing lower plasma levels of pregnenolone, DHEA, and DHEA-sulfate compared to younger women [73]. Consequently, several studies have reported interval references for individual steroid compounds to reduce the risk of misinterpretation and inappropriate clinical decisions [75,84,85]. Therefore, including standardized reference intervals in steroid studies should be considered to facilitate the translation of steroidomics to clinical practice [84].

Regarding the steroid profiling method, MS-based methods are the current state-of-the-art analytical platforms. These methods allow for high-throughput analysis and provide more specific qualitative and quantitative steroid metabolism. It should be noted that both GC-MS and LC-MS have their own advantages and disadvantages. Therefore, GC-MS and LC-MS should be complementary approaches to comprehensively detect steroid compounds in biological matrixes [86]. The statistical methods are also worth noting here; they have been discussed at length in a previous report by our group [70]. Currently, there have been no studies that have integrated multi-layer omics data, such as transcriptomics and proteomics, with steroidomics for cancer risk assessment, diagnosis, and prognosis. Moreover, the utility of statistical learning has not been thoroughly tested. These two complementary approaches should be considered as potential topics of research in the future. It should be noted that we, along with other researchers, have discussed and demonstrated benefits and pitfalls when applying statistical modeling in omics-related cancer research [87,88,89,90,91,92]. Furthermore, there is a demand for standardized and calibrated analytical procedures and new analytical methods to characterize the steroid metabolome [84,93,94]. The integration of multidimensional omics data has achieved considerable success, and there is still room for improvement, especially in the construction of multi-omics prediction models [95,96,97].

Estrogens and estrogen metabolites were the central molecules in our network visualization. The network exhibited the connection between the estrogen and estrogen metabolites together with the associated enzymes. This observation is in accordance with the role of estrogens in cancer. Additionally, it suggests candidates for discovering new cancer therapeutics based on steroidogenic enzymes, which were recently investigated by others [98,99]. Regarding other differentially expressed steroids, estradiol, DHEA, and cortisol were the most promising among steroid biomarkers. In particular, estradiol was associated with an increased risk of BC and EC, but with a decreased risk of E/GC [40,42,50]. Positive DHEA supplementation effects have been demonstrated in many disease stages [100]. In fact, DHEA has been shown to have a protective role against the proliferation and migration of breast and cervical cancers [101,102]. Notably, Petrick and colleagues reported that the increase in DHEA plasma level significantly lowered the risk of esophageal/gastric cardia adenocarcinoma [40]. However, the mechanism of this beneficial effect is not well explored. High nocturnal cortisol level is correlated with the short-term survival of epithelial ovarian cancer patients [21]. In addition, other commonly reported steroids, such as testosterone, androstenedione, and DHEA-sulfate, were also observed to predict increased risk of breast cancer in women, with the elevation of premenopausal serum [103]. Generally, steroidogenesis has been extensively targeted by using inhibitors of steroidogenesis as therapeutic agents for cancer and other diseases [104,105,106]. Recently, the underlying mechanism of steroidogenesis was characterized in castration-resistant prostate cancer cell lines and tissues to provide better insights into steroid substrate utilization [107]. Moreover, differential gene expressions related to androgen and estrogen metabolism were explored between hereditary and sporadic prostate cancer [108]. In addition, estrogen and androgen blockade is a potential direction for personalized medicine in PC [109]. Androstenedione is a precursor of several steroids, such as testosterone, estradiol, and estrone. Therefore, catabolism of androstenedione might be linked to cancer-related androgen and estrone metabolism. Particularly, inhibiting the indirect conversion of androstenedione to dihydrotestosterone might help reduce the risk of PC [110].

## 4. Materials and Methods

### 4.1. Systematic Literature Search Strategy

This study was conducted in accordance with the Preferred Reporting Items for Systematic Reviews and Meta-Analyses (PRISMA, Table S6) guidelines [111]. Systematic literature searches were performed on PubMed, Scopus, Embase, Web of Science Core Collection, Virtual Health Library (VHL), and Cochrane Controlled Register of Trials (CENTRAL) using the keywords: (steroid OR steroids) AND (tumor OR tumors OR tumour OR tumours OR tumorous OR tumourous OR malignancy OR cancer OR cancers OR cancerous OR carcinoma OR carcinoma OR adenoma OR neoplasm OR neoplasms OR neoplastic OR neoplasia) AND (diagnosis OR treatment OR therapy OR prognosis OR recurrence) AND (“metabolite profiling” OR “metabolite analysis” OR “metabolic profiling” OR “metabolic fingerprinting” OR “metabolic characterization” OR metabolite OR metabolome OR metabolomics OR metabolomic OR metabonomics OR metabonomic). The search results were first retrieved in March 2018 and updated regularly up to February 2019. This was supplemented with a manual search of bibliographic reference lists to include all relevant studies. There was no limit regarding the publication period.

### 4.2. Inclusion and Exclusion Criteria

The search results were retrieved from the online databases and imported into Endnote X8 software. After the removal of duplicate records, the remaining records were imported to Rayyan, a tool for systematic reviews [112]. Next, we assessed the eligibility of each article by evaluating the title and abstract. The articles suited for the next step were selected if they met the following inclusion criteria: (1) reported at least one steroid molecule as a novel marker in patients with any cancers; (2) had comparison groups (cancer versus healthy/non-cancerous patients, before and after treatment); (3) used any high-throughput techniques including liquid chromatography-mass spectrometry (LC-MS), gas chromatography-mass spectrometry (GC-MS), nuclear magnetic resonance (NMR); (4) performed statistical biomarker selection and evaluation (univariate, multivariate analysis, or machine learning) for the prediction, discrimination, and management of cancers from healthy and/or non-cancerous conditions. On the other hand, studies were excluded using the following criteria: (1) were in vitro cell culture or non-human studies; (2) had no suitable control groups; (3) were case reports and series, proposals, letters, conference abstracts, proposals, meeting records, or review articles; (4) overlapped population but had smaller sample size; (5) had no suitable analytical platforms; and (6) had no available abstract or full text. All references were independently assessed for inclusion or exclusion by at least two authors to avoid personal bias. The final included articles were then classified into four categories by the primary purpose of the study: risk prediction, diagnosis, prognosis, and therapy monitoring.

### 4.3. Data Extraction

The protocol of data extraction was according to that previously published by our group [70]. Briefly, the details on study design and population characteristics (year, sample size, age, gender, tumor type, tumor stage, reference diagnostic method, hormone therapy, and follow-up) were recorded from each eligible paper. Next, information relating to biospecimens (serum, plasma, urine), instrumental platforms such as GC-MS and LC-MS, fasting condition, sample preparation, sample storage, internal standard, analytical validation, compound identification method, and outlier detection was also recorded. Finally, details on the alterations of steroid compounds, statistical analyses, and if applicable, reference ranges of each article were noted. The data extractions were carried out independently by at least two review authors (NHA and SJK). Cholesterol and vitamin D were not considered as steroid molecules in this study.

### 4.4. Quality Assessment of Included Studies

QUADOMICS, an adaptation of the Quality Assessment of Diagnostic Accuracy Assessment, was conducted to assess the quality of each included study [113]. The items 2 and 14, only applied for studies in phase 4, were not utilized in our study. In addition, the remaining 14 items were scored as “not available” if it could not be applied to a study. All study quality assessments were conducted independently by at least two authors (NHA and NPL), to avoid personal bias.

### 4.5. Steroid Functional Analysis and Pathway Visualization

The steroids reported in at least two studies were abstracted and included in the steroid metabolism pathway. We extracted the steroid nomenclature from Human Metabolome Database (HMDB) version 4.0 and then performed pathway enrichment analysis and network analysis using Metaboanalyst version 4.0 and OmicsNet, respectively [114,115,116]. The Small Molecular Pathway Database (SMPD) was used as the knowledgebase for conducting relevant enrichment analysis [117]. Other parameters were set as default. For accurate identification and classification of the steroids, we supplemented the nomenclature reported in the included studies by applying HMDB identification (HMDB ID) for the analysis. Compounds without HMDB IDs were excluded before the pathway and network analysis.

## 5. Conclusions

Oncosteroidomics is a promising approach for clinical cancer research. Along with remarkable achievements at the initial stage, more efforts are needed to establish standardized methods to utilize the alterations of steroid metabolic networks for the prevention, assessment, and management of cancers. Rigorous study design, sampling procedure, analytical approaches, and statistical methods are required to provide more insightful applications of steroidomics in the future.

## Figures and Tables

**Table 1:** Demographic characteristics of the included studies.

Study and Year of Publication	Sample Collection	Cohort Allocation	Aim	Patients							Controls					Follow-Up
				Type	Diagnosis	No.	Age	M/F	Stage	Hormone Treatment	Type	Match	No.	Age	M/F	
Schweitzer et al. (2018) [26]	Prospective	ENSAT	Diagnosis	ACC	Pathologically confirmed	42	M: 57; R: 20–80	15/27	I-V	NA	ACA	Yes	66	M: 58; R: 21–81	29/37	No
Hines et al. (2017) [27]	Prospective	US	Diagnosis	ACC	Pathologically confirmed	5	NA	NA	NA	No	H, ACA	No	114, 61	M^1^: 42, 47; R^1^: 24–83, 25–83	48/66	NA
Taylor et al. (2017) [28]	Prospective	UK	Diagnosis	ACC	Pathologically confirmed	10	M: 59; R: 47–69	4/6	NA	NA	ACA, PPC/PGL, NFAA	Yes	7, 15, 16	M: 68, 50, 62; R: 66–70, 44–66, 48–72	4/3; 8/7; 6/10	NA
Qian et al. (2016) [29]	Prospective	China	Diagnosis	Primary LC	AJCC	66	m: 57.5; SD: 9.6	66/0	I-II	No	CL, H	No	59, 65	m: 50.6, 53.6; SD: 12.5, 15.4	59/0; 65/0	NA
Velikanova et al. (2016) [30]	Prospective	Russia	Diagnosis	ACC	Pathologically confirmed	31	M: 43; R: 33–57	8/23	NA	Yes	ACA-HNA, ACA-CS, H	No	52, 44, 25	M: 55, 48; R: 50–61, 21–54	17/35; 18/26	NA
Kerkhofs et al. (2015) [20]	Retrospective	Netherland	Diagnosis	ACC	Pathologically confirmed^2^	27	m: 57; SD: 14	8/19	II-IV	NA	ACA function, ACA non function	No	22, 85	m: 50, 58; SD: 12, 12	6/16; 28/57	Yes
Dai et al. (2014) [31]	Prospective	China	Diagnosis	HCC	Pathologically confirmed	28	NA	NA	I^3^	NA	H, CL	NA	21, 21	NA	NA	NA
Perna et al. (2014) [32]	Prospective	UK	Diagnosis	ACC	Pathologically confirmed	13	m: 51.7; SD: 16.2	4/9	NA	NA	ACA-RML, ACA	No	7, 11	m: 70.14, 54.3; SD: 8.84, 12.35	4/3; 2/9	NA
Konieczna et al. (2013) [33]	Prospective	Poland	Diagnosis	BlC, KC, PC, TC, others	Pathologically confirmed	58, 11, 9, 3, 11^4^	m: >40	46/12; 7/4; NA; NA; NA	NA	NA	H	No	100	m: >40	61/39	NA
Konieczna et al. (2013) [23]	Prospective	Poland	Diagnosis	BlC, KC, PC, TC, others	NA	47, 10, 7, 3, 10^4^	m: 65.00; SD: 10.40	17/60	NA	No	H	Yes	77	m: 46.97; SD: 18.51	38/39	NA
Arlt et al. (2011) [34]	Retrospective	ENSAT	Diagnosis	ACC	Pathologically confirmed	45	M: 55; R: 20–80	24/21	NA	No	ACA, H	NA	102, 88	M: 60; R: 19–84; 18–60	39/63; 26/62	Yes
Bufa et al. (2010) [35]	Prospective	Hungary	Diagnosis	AE	NA	13	m: 67.9; SD: 8.5	0/13	NA	NA	H	Yes	10	m: 58.7; SD: 6.2	0/10	NA
Bufa et al. (2008) [36]	Prospective	Hungary	Diagnosis	EOC	NA	15	m: 60.4; SD: 5.1	0/15	NA	NA	H	Yes	10	m: 58.7; SD: 6.2	0/10	NA
Drafta et al. (1982) [37]	Prospective	Romania	Diagnosis	PC	UICC 1974 and VACRG	32	m: 67; R: 51–79	32/0	I-IV	NA	BPH, H	Yes^5^	54, 63	m: 68, 66; R: 50–78, 50–79	54/0; 63/0	NA
Trabert et al. (2019) [38]	Retrospective	WHI-OS	Risk prediction	OC	NA	169	m: 64.1; SD: 7.2	0/169	NA	No	H	Yes	410	m: 64.3; SD: 7.2	0/410	Yes
Petrick et al. (2018) [39]	Retrospective	Northern Ireland, Ireland	Risk prediction	EA	Pathologically confirmed	172	m: 64.3; SD: 10.9	172/0	NA	No	H	Yes	185	m: 63.5; SD: 12.6	185/0	NA
Petrick et al. (2018) [40]	Retrospective	PLCO, ATBC, CPS-II nutrition cohort	Risk prediction	EA/GCA	NA	259	m: 62.0; SD: 6.6	259/0	NA	No	H	Yes	259	m: 61.0; SD: 6.6	259/0	NA
Sampson et al. (2017) [41]	Retrospective	PLCO, US, B-FIT, SWHS	Risk prediction	BC	NA	1298	NA	0/1298	NA	No	H	Yes	1524	NA	0/1524	Yes
Brinton et al. (2016) [42]	Retrospective	WHI-OS	Risk prediction	EC	NA	313	m: 64.5; SD: 7.0	0/313	NA	No	H	Yes	354	m: 64.0; SD: 7.0	0/354	Yes
Moore et al. (2016) [43]	Retrospective	China	Risk prediction	BC	NA	399	NA	0/399	NA	No	H	Yes	399	NA	0/399	Yes
Trabert et al. (2016) [44]	Retrospective	WHI-OS	Risk prediction	OC	NA	169	m: 64.1; SD: 7.2	0/169	NA	No	H	Yes	412	m: 64.3; SD: 7.2	0/412	Yes
Dallal et al. (2016) [45]	Retrospective	B-FIT	Risk prediction	EC	NA	66	m: 67.5; SD: 5.6	0/66	NA	No	H	No	346	m: 67.0; SD: 6.2	0/346	Yes
-	Retrospective	B-FIT	Risk prediction	OC	NA	67	m: 68.5; SD: 5.7	0/67	NA	No	H	No	416	m: 67.0; SD: 6.3	0/416	Yes
Schairer et al. (2015) [46]	Retrospective	PLCO	Risk prediction	BC (estrogen receptor positive)	NA	193	R: 55–74	0/193	NA	No	H	Yes	268	NA	0/268	Yes
Black et al. (2014) [47]	Retrospective	PLCO	Risk prediction	PC	NA	195	R: 55–70	195/0	III-IV	No	H	Yes	195	R: 55–70	195/0	Yes
Falk et al. (2013) [48]	Retrospective	US	Risk prediction	BC	NA	215	NA	0/215	NA	No	H	Yes	215	NA	0/215	Yes
Dallal et al. (2013) [49]	Retrospective	B-FIT	Risk prediction	BC	NA	407	m: 67.2; SD: 5.7	0/407	NA	No	H	No	496	m: 67.3; SD: 6.2	0/496	Yes
Fuhrman et al. (2012) [50]	Retrospective	PLCO	Risk prediction	BC	NA	277	R: 55–74	0/277	NA	No	H	No	423	R: 55–74	0/423	Yes
Audet-Walsh et al. (2010) [51]	Retrospective	Canada	Risk prediction	EC	NA	126	m: 64.8; SD: 9.1	0/126	I-IV	No	H	No	110	m: 58.3; SD: 5.6	0/110	NA
Yang et al. (2009) [52]	Prospective	US	Risk prediction	PC	NA	14	m: 63.6; R: 50–83	14/0	NA	NA	H	No	125	m: 64.8; R: 45–78	125/0	NA
Lévesque et al. (2019) [53]	Retrospective	Canada	Prognosis	PC	NA	1776^6^	m: 62.7; SD: 6.4	1776/0	I-IV	No	PC	Yes	1776^6^	m: 62.7; SD: 6.4	1776/0	Yes
Audet-Delage et al. (2018) [54]	Prospective	Canada	Prognosis	EC	Pathologically confirmed	246	m: 65.1; SD: 8.9	0/246	I-IV	No	EC^7^, H	Yes	246, 110	m: 65.1, 58.3; SD: 8.9, 5.6	0/246; 0/110	Yes
Plenis et al. (2013) [55]	Prospective	Poland	Prognosis	NET	NA	19^8^	m: 54.6; SD: 11.8	10/9	NA	NA	H	Yes	20	m: 47.3; SD: 12.5	10/10	NA
Lévesque et al. (2013) [56]	Prospective	Canada	Prognosis	PC	Pathologically confirmed	526^9^	m: 63.3; SD: 6.8	NA	NA	No	NA	NA	NA	NA	NA	NA
Thomas et al. (1982) [57]	Prospective	UK	Prognosis	BC^10^	Pathologically confirmed	109	NA	0/109	I-II	NA	BC^11^	NA	109	NA	0/109	Yes
Zang el at. (2014) [58]	Prospective	US	Diagnosis	PC	NA	64	m: 59; R: 49–65	64/0	NA	No	H	Yes	50	m: 50; R: 45–76	50/0	NA
Song et al. (2012) [59]	Prospective	China	Diagnosis	GC	Pathologically confirmed	30	M: 63; R: 39-88	15/15	I-IV	No	H	Yes	30	M: 62; R: 42–82	15/15	No
Moore et al. (2018) [60]	Retrospective	PLCO	Risk prediction	BC	NA	621	R: 55–74	0/621	NA	No	H	Yes	621	R: 55–74	0/621	Yes
Huang et al. (2017) [61]	Retrospective	Finland	Risk prediction	PC	NA	137	m: 59.8, 58, 60.9	137/0	II-IV	NA	H	Yes	200	m: 59.3	200/0	NA
Mondul et al. (2015) [62]	Retrospective	ATBC	Risk prediction	PC	AJCC	200	m: 59.4	200/0	III-IV	No	H	Yes	200	m: 59.3	200/0	Yes
Huang et al. (2018) [63]	Retrospective	Finland	Prognosis	3rd tertile of PC	AJCC	197^6, 12^	m: 69; R: 55–86	197/0	I-IV	NA	1st and 2nd tertile of PC	No	197^6, 12^	m: 69; R: 55–86	197/0	Yes
Ye et al. (2014) [64]	Prospective	China	Prognosis	OSCC (S)	UICC 2002	11	M: 52; R: 35–74	7/4	III-IVA	No	OSCC (NS)	Yes	21	M: 53; R: 45–71	15/6	NA
Zhou et al. (2014) [65]	Prospective	China	Prognosis	HCC	6^th^ TNM	22^13^	m: 47; SD: 12	19/3	I-IIIB^14^	NA	HCC	Yes	18	m: 45; SD: 11	15/3	Yes
Miller et al. (2015) [66]	Prospective	US	Therapy monitoring	BC after limonene intervention	Pathologically confirmed	40^6^	M: 58.5; IQR: 18.5	0/40	IS-T1	NA	BC before limonene intervention	Yes	40^6^	M: 58.5, IQR: 18.5	0/40	NA
Ghataore et al. (2012) [67]	Prospective	France	Therapy monitoring	ACC	Pathologically confirmed	17	M^15^: 50/47; R^15^: 26–66/20–76	6/11^16^	NA	Yes	H	No	40	M^15^: 31/29; R^15^: 22–49/20–59	20/20	Yes
Saylor et al. (2012) [68]	Prospective	US	Therapy monitoring	PC after ADT	NA	36	NA	36/0	NA	No	PC before ADT	Yes	36	NA	36/0	Yes

AJCC: American Joint Committee on Cancer; ACC: Adrenocortical carcinoma; ACA: Adrenocortical adenoma; ADT: Androgen deprivation therapy; AE: Adenocarcinoma endometrii; ATBC: The Alpha-Tocopherol, Beta-Carotene Cancer Prevention; ACA-CS: Adrenocortical adenoma with Cushing’s syndrome; ACA-HNA: Adrenocortical adenoma hormonally non-active adenomas; ACC-RML: Adrenocortical adenoma with regression and myelolipomatous changes; BC: Breast Cancer; BPH: Benign protatic hyperplasia; BlC: Bladder cancer; B-FIT: The Breast and Bone Follow-up to the Fracture Intervention Trial; CL: Cirrhotic liver; CSP II: Cancer Prevention Study II; EA: Esophageal adenocarcinoma; EOC: Epithelial ovarian cancer; ENSAT: European Network for the Study of Adrenal Tumors; EC: Endometrial cancer; GC: Gastric cancer; GAC: Gastric cardia adenocarcinoma; H: Health; HCC: Hepatocellular carcinoma; IQR: Interquartile range; KC: Kidney cancer; LC: Liver cancer; M: Median; m: Mean; NET: Neuroendocrine Tumor; NFAA: Nonfunctioning adrenal adenoma; NA: Not available; OSCC (S): Oral squamous cell carcinoma patient with significant chemotherapy efficacy; OSCC (NS): Oral squamous cell carcinoma patient with nonsignificant chemotherapy efficacy; PC: Prostate cancer; PCC/PGL: Phaeochromocytoma/paraganglioma; PLCO: The Prostate, Lung, Colorectal and Ovarian; OC: Ovarian cancer; R: Range; SD: Standard deviation; SWHS: The Shanghai Women’s Health Study; TC: Testis cancer; TNM: TNM Classification of Malignant Tumors; UICC: Union for International Cancer Control; VACURG: Veterans Administration Cooperative Urologic Research Group; WHI-OS: Women’s Health Initiative Observational Study; 1: From healthy male and female individuals; 2: 25/27 patients; 3: 24/28 patients; 4: Patients in other urogenital tract cancers; 5: PC match with the control group; 6: Total population; 7: Sample taken after surgery; 8: Patients treated with somatostatin analogs; 9: Postmenopausal population; 10: BC with lower androsterone and aetiocholonolone levels than medium; 11: BC with higher androsterone and aetiocholonolone levels than medium; 12: 92 prostate cancer deaths during the period of follow-up; 13: Early intrahepatic recurrence; 14: Stage for both groups; 15: Value of male/female; 16: Six of premenopausal age.

**Table 2:** Potential steroid biomarkers ^*^ reported in at least four studies.

Steroid Compound	Biomarker Function in Cancer											Reference
	ACC	PC	BC	BlC	EC	LC	KC	TC	NET	OC	E/GC	
Estradiol	↑	↑	↑		↑	↑				↑	↓	[26,29,37,39,40,41,42,43,44,45,46,48,49,50,54]
Dehydroepiandrosterone	↑	↑			↑↓	↑				↑	↓	[26,27,30,32,36,39,40,51,53,54,56]
Cortisol	↑	↑		↑		↓			→			[23,26,27,28,30,31,33,37,55,68]
Pregnanetriol	↑	↑			↑					↑		[20,27,30,34,35,36]
Testosterone		↑		↓	↑	↓	↑		→		↓	[23,29,33,37,39,51,54,55,56]
Estrone		↑	↑		↑↓	↑				↑	↓	[29,37,39,41,42,43,44,48,51,54]
2-methoxyestrone		↑	↑		↑					↑		[41,42,43,44,47,49]
Pregnanediol	↑									↑		[20,27,30,32,34,36]
Androsterone	↑	↑	↓		↑↓						↓	[20,35,39,54,56,57]
Dehydroepiandrosterone sulfate	↑	↑			↑	↑						[26,28,51,53,54,65,68]
2-hydroxyestrone		↑	↑		↑					↑		[41,42,43,44,45,48]
Estriol			↑		↑					↑		[41,42,43,44,48,49,54]
16-epiestriol			↑		↑					↑		[41,42,43,44,48,49]
16α-hydroxyestrone			↑		↑					↑		[41,42,43,44,45]
Etiocholanolone	↑		↑		↑							[20,27,30,34,35,57]
Androstenedione	↑	↑			↑							[26,28,51,54,58]
Dihydrotestosterone	↑	↑			↑						↓	[26,39,51,54,56]
16-ketoestradiol			↑		↑					↑		[41,42,43,44,49]
Tetrahydrodeoxycortisol	↑											[20,27,30,34]
Cortisone	↑			↑					→			[23,27,30,33,55]
Progesterone	↑	↓		↓			↓	↓	→			[23,26,33,55]
Androstenediol					↑						↓	[39,51,53,54]
2-hydroxyestrone-3-methyl ether			↑		↑							[41,42,43,48]
4-hydroxyestrone			↑		↑					↑		[41,42,43,44,45]
4-methoxyestrone		↑			↑					↑		[42,44,47,54]
17-epiestriol			↑		↑							[41,42,43,45,49]

↑: Significant increase in cancer or more aggressive group; ↓: Significant decrease in cancer or more aggressive group; →: Significant differences (not shown if increased or decreased); * The order of steroids was sorted based on number of papers recorded; AC: Adrenal cancer; PC: Prostate cancer; BC: Breast cancer; BlC: Bladder cancer; EC: Endometrial cancer; LC: Liver cancer; KC: Kidney cancer; TC: Testicle cancer; NET: Neuroendocrine Tumor; OC: Ovarian cancer; E/GC: Esophagus/Gastric cancer.

## Supplementary Materials

The following are available online at https://www.mdpi.com/2218-1989/9/10/199/s1, Figure S1: Descriptive summary of the included studies. (a) The number of included studies in each year, (b) type of cancer, (c) study purpose, Figure S2: Pathway visualization of the two steroid pathways altered in cancers. (a) Androgen and Estrogen Metabolism, (b) Androstenedione Metabolism. The red boxes refer to the potentially altered steroids in the included studies, Table S1: Study designs and metabolomics approaches of the included studies, Table S2: The key findings and steroid biomarkers in the included studies, Table S3: The QUADOMICS quality assessment results of the included studies, Table S4: The steroids reported in the included studies, Table S5: The characteristics of significantly enriched pathways analysis, Table S6: PRISMA checklist.
