# Supplementary material for: Steroidomics for the Prevention, Assessment, and Management of Cancers: A Systematic Review and Functional Analysis
Source: Metabolites. 2019 Sep 21;9(10):199. doi: 10.3390/metabo9100199 (PMC6835899; doi:10.3390/metabo9100199)

SUPPLEMENTARY MATERIALS

Steroidomics for the prevention, assessment, and management of cancers: A systematic review and functional analysis

Nguyen Hoang Anh [1,†], Nguyen Phuoc Long [1,†], Sun Jo Kim [1], Jung Eun Min [1], Sang Jun Yoon [1], Hyung Min Kim [1], Eugine Yang [2], Eun Sook Hwang [2], Jeong Hill Park [1], Soon-Sun Hong [3], Sung Won Kwon [1,*]

1 College of Pharmacy, Seoul National University, Seoul 08826, Republic of Korea.
2 College of Pharmacy, Ewha Womans University, Seoul 03760, Republic of Korea.
3 Department of Biomedical Sciences, College of Medicine, Inha University, Incheon 22212, Republic of Korea.
† These authors contributed equally to this work.
* Correspondence: Sung Won Kwon (swkwon@snu.ac.kr)

Table S1. Study designs and metabolomics approaches of the included studies.

Study	Steroidomics	Platforms	Specimen	Fasting condition	Sample storage	Sample preparation	Reference Interval	Internal standards	Compound identification	Analytical validation
**Schweitzer *et al.* (2018) [1]**	Targeted steroidomics	LC-MS	Plasma	No	-80 °C	Yes	ND	Yes	Commercial kit	QC
**Hines *et al.* (2017) [2]**	Targeted steroidomics	LC-MS	Urine	No	-70 °C	Yes	Yes	Yes	Authentic standards	QC
**Taylor *et al.* (2017) [3]**	Targeted steroidomics	LC-MS	Serum	No	-20 °C	Yes	Yes	Yes	Authentic standards	QC
**Qian *et al.* (2016) [4]**	Targeted steroidomics	LC-MS	Serum	No	-40 °C	Yes	ND	Yes	Authentic standards	QC
**Velikanova *et al.* (2016) [5]**	Targeted steroidomics	LC-DAD & GC-MS	Urine	No	NA	Yes	ND	ND	ND	ND
**Kerkhofs *et al.* (2015) [6]**	Targeted steroidomics	GC-MS	Urine	No	NA	NA	Yes	Yes	ND	ND
**Dai *et al.* (2014) [7]**	Targeted steroidomics	LC-MS	Urine	No	-80 °C	Yes	ND	ND	In-house library	ND
**Perna *et al.* (2014) [8]**	Targeted steroidomics	GC-MS	Urine	No	NA	Yes	ND	Yes	ND	ND
**Konieczna *et al.* (2013) [9]**	Targeted steroidomics	LC-MS	Urine	No	NA	Yes	ND	Yes	Authentic standards	ND
**Konieczna *et al.* (2013) [10]**	Targeted steroidomics	LC-MS	Urine	No	NA	Yes	ND	Yes	Authentic standards	QC
**Arlt *et al.* (2011) [11]**	Targeted steroidomics	GC-MS	Urine	No	-20 °C	Yes	Yes	ND	ND	ND
**Bufa *et al.* (2010) [12]**	Targeted steroidomics	GC-MS	Urine	No	-20 °C	ND	Yes	Yes	Authentic standards	ND
**Bufa *et al.* (2008) [13]**	Targeted steroidomics	GC-MS	Urine	No	-20 °C	Yes	ND	Yes	Authentic standards	ND
**Drafta *et al.* (1982) [14]**	Targeted steroidomics	Open column[1]	Plasma	No	-20 °C	ND	ND	ND	ND	ND
**Trabert *et al.* (2019) [15]**	Targeted steroidomics	LC-MS	Serum	Yes	-70 °C	Yes	ND	Yes	Authentic standards	ND

**Petrick** *et al.* **(2018) [16]**	Targeted steroidomics	GC-MS	Plasma	No	-80 °C	Yes	ND	ND	ND	QC
**Petrick** *et al.* **(2018) [17]**	Targeted steroidomics	GC-MS	Serum	Yes[2]	-70 °C[3]; -130 °C[4]	ND	ND	Yes	ND	QC
**Sampson** *et al.* **(2017) [18]**	Targeted steroidomics	LC-MS	Serum	Yes	-70 °C	ND	ND	Yes	Authentic standards	QC
	Targeted steroidomics	LC-MS	Urine	Yes	-80 °C	ND	ND	Yes	Authentic standards	QC
**Brinton and Trabert** *et al.* **(2016) [19]**	Targeted steroidomics	LC-MS	Serum	Yes	-70 °C	Yes	ND	Yes	Authentic standards	QC
**Moore** *et al.* **(2016) [20]**	Targeted steroidomics	LC-MS	Urine	Yes	-80 °C	ND	ND	Yes	Authentic standards	QC
**Trabert** *et al.* **(2016) [21]**	Targeted steroidomics	LC-MS	Serum	Yes	-70 °C	Yes	ND	Yes	Authentic standards	QC
**Dallal** *et al.* **(2016) [22]**	Targeted steroidomics	LC-MS	Serum	No	-20 °C[5]	Yes	ND	Yes	Authentic standards	QC
**Schairer** *et al.* **(2015) [23]**	Targeted steroidomics	LC-MS	Serum	No	-80 °C	Yes	ND	Yes	Authentic standards	QC
**Black** *et al.* **(2014) [24]**	Targeted steroidomics	LC-MS	Serum	No	-70 °C	Yes	ND	Yes	Authentic standards	QC
**Falk** *et al.* **(2013) [25]**	Targeted steroidomics	LC-MS	Serum	ND	-70 °C	ND	ND	Yes	Authentic standards	QC
**Dallal** *et al.* **(2013) [26]**	Targeted steroidomics	LC-MS	Serum	No	-20 °C[5]	Yes	ND	Yes	Authentic standards	QC
**Fuhrman** *et al.* **(2012) [27]**	Targeted steroidomics	LC-MS	Serum	No	-80 °C	Yes	ND	Yes	Authentic standards	QC
**Audet-Walsh** *et al.* **(2010) [28]**	Targeted steroidomics	GC-MS & LC-MS	Serum	No	-80 °C	Yes	ND	Yes	Authentic standards	QC
**Yang** *et al.* **(2009) [29]**	Targeted steroidomics	LC-MS	Urine	No	-80 °C	Yes	ND	ND	Authentic standards	ND
**Lévesque** *et al.* **(2019) [30]**	Targeted steroidomics	GC-MS & LC-MS	Plasma	No	-80 °C	ND	ND	Yes	Authentic standards	QC
**Audet-Delage** *et al.* **(2018) [31]**	Targeted steroidomics	GC-MS & LC-MS	Serum	No	-80 °C	Yes	ND	Yes	Authentic standards	QC

**Plenis *et al.* (2013) [32]**	Targeted steroidomics	LC-UV	Urine	No	-80 °C	Yes	ND	Yes	Authentic standards	QC
**Lévesque *et al.* (2013) [33]**	Targeted steroidomics	GC-MS	Plasma	Yes	-80 °C	Yes	ND	Yes	Authentic standards	ND
**Thomas *et al.* (1982) [34]**	Targeted steroidomics	GC-FID	Urine	No	-20 °C	ND	ND	ND	ND	ND
**Zang *et al.* (2014) [35]**	Untargeted metabolomics	LC-MS	Serum	No	-80 °C	ND	NA	ND	In-house library	ND
**Song *et al.* (2012) [36]**	Untargeted metabolomics	GC-MS	Serum	Yes	-20 °C	Yes	NA	ND	In-house library	ND
**Moore *et al.* (2018) [37]**	Untargeted metabolomics	LC-MS	Serum	No	-70 °C	Yes	NA	Yes	Authentic standards	QC
**Haung *et al.* (2017) [38]**	Untargeted metabolomics	GC-MS & LC-MS	Serum	Yes	-70 °C	No	NA	ND	ND	QC
**Mondul *et al.* (2015) [39]**	Untargeted metabolomics	GC-MS & LC-MS	Serum	Yes	-70 °C	No	NA	ND	In-house library	QC
**Huang *et al.* (2018) [40]**	Untargeted metabolomics	GC-MS & LC-MS	Serum	Yes	-70 °C	ND	NA	ND	In-house library	QC
**Ye *et al.* (2014) [41]**	Pseudotargeted (semitargeted) metabolomics	GC-MS	Serum	Yes	-80 °C	Yes	NA	Yes	Authentic standards & in-house library	QC
**Zhou *et al.* (2014) [42]**	Untargeted metabolomics	LC-MS	Plasma	No	-80 °C	Yes	NA	ND	ND	QC
**Miller *et al.* (2015) [43]**	Untargeted metabolomics	GC-MS & LC-MS	Plasma	ND	-80 °C	Yes	NA	Yes	In-house library	QC
**Ghataore *et al.* (2012) [44]**	Untargeted metabolomics	GC-MS	Urine	No	ND	ND	NA	Yes	Authentic standards	ND
**Saylor *et al.* (2012) [45]**	Untargeted metabolomics	GC-MS & LC-MS	Plasma	Yes	-70 °C	Yes	NA	Yes	In-house library	QC

1: Sephadex LH-20

2: Fasting at least 12h in ATBC trial

3: PLCO trial and ATBC trial

4: CPS-II Nutrition Cohort

5: Sample was stored at -20 ^oC for three years and then moved to -70 ^oC

GC-MS Gas chromatography-mass spectrometry; LC-MS: Liquid chromatography-mass spectrometry; GC-FID: Gas Chromatography-Flame Ionization Detector; LC-DAD: Liquid Chromatography With Diode Array Detection

Table S2. The key findings and steroid biomarkers in the included studies.

Study	Statistics	Additional factor	Normalization method	Steroids significant alteration	Key findings
**Schweitzer *et al.* (2018) [1]**	Wilcoxon rank sum test	NA	NA	Progesterone; 17-hydroxyprogesterone; 11-deoxycortisol; DHEA; DHEA-S; estradiol; 11-deoxycorticosterone; cortisol	The concentration is significantly higher in ACC patients compared to ACA.
	Logistic regression modeling	NA	NA	Corticosterone; estradiol; progesterone; 11-deoxycortisol; DHEA; 4-dione	The model of six steroids showed the best predictive parameters in males with AIC of 34.48 and AUC of 0.95.
	Logistic regression modeling	NA	NA	DHEA-S; 4-dione; DHEA; DHT; 11-deoxycorticosterone; aldosterone	The model of six steroids showed the best predictive parameters in females with AIC of 50.68 and AUC of 0.94.
**Hines *et al.* (2017) [2]**	Student t-tests or Wilcoxon/ Kruskal–Wallis tests	NA	Total urine volume	Etio; DHEA; 5PT; 5PD; pregnanediol; 17HP; pregnanetriol; THS; cortisol; 6β-hydroxycortisol; cortisone	Significantly higher in patients with ACC compared to ACA.
**Taylor *et al.* (2017) [3]**	Pairwise Mann–Whitney U-tests; with post hoc Bonferroni correction	NA	NA	4-dione; DHEA-S; 17-hydroxyprogesterone; 17HP; 11-deoxycortisol	Significantly higher in ACC compared to cortisol-producing adenoma group.
	Pairwise Mann–Whitney U-tests; with post hoc Bonferroni correction	NA	NA	4-dione; 17-hydroxyprogesterone; 17HP; 11-deoxycortisol; 11-deoxycorticosterone; cortisol	Significantly higher in ACC compared to PPC/PGL group.

	Pairwise Mann–Whitney U-tests; with post hoc Bonferroni correction	NA	NA	4-dione; 17-hydroxyprogesterone; 17HP; 11-deoxycortisol; 11-deoxycorticosterone; cortisol	Significantly higher in ACC compared to NFAA group.
**Qian *et al.* (2016) [4]**	Mann-Whitney U test	NA	NA	Estradiol; estrone; testosterone	The steroids are significantly different for the three groups. Estradiol and estrogen level is higher in primary LC compared to liver cirrhosis and healthy group while lower testosterone level is observed in primary liver cancer and liver cirrhosis compared to the healthy group.
	PLS-DA	NA	NA	Estradiol/testosterone and (estradiol+ estrone)/testosterone	The model distinguished between three groups (liver cancer, cirrhosis, and healthy with R2Y:0.701 and Q2Y:0.583).
	ROC analysis	NA	NA	(Estradiol+estrone)/testosterone	The combination of (estradiol+estrone)/testosterone and AFP improved specificity and diagnostic accuracy with AUC of 0.862 compared to (estradiol+estrone)/testosterone and AFP alone
**Velikanova *et al.* (2016) [5]**	Mann-Whitney U Test	NA	NA	Free cortisol; free cortisone; pregnanediol; THE; THB; THF; a-THF; α + β-cortolones; α + β-cortols; dihydrocortisol; cortisol	Significantly higher in ACA-CS.
	Mann-Whitney U Test	NA	NA	6β-hydroxycortisol/free cortisol ratio; DHEA	Significantly lower in ACA-CS compared to ACC-HNA.
	Mann-Whitney U Test	NA	NA	6β-hydroxycortisol; 6β-hydroxycortisol/free cortisol ratio; Etio; androstendiol-17β; DHEA; 16-hydroxy-DHEA; 11-hydroxy-etiocholanolone; androstenetriol; 16-oxo-androstendiol; pregnanediol; pregnanetriol; 11-oxo-pregnanetriol; pregnenediol; 5-pregnen,3α,l16α,20α-triol; 5-pregnen,3α,17α,20α-triol; 6-hydroxy-pregnanolone; THS; hexahydro-11-deoxycortisol; 21-deoxy-	The metabolites were significantly higher in ACC compared to ACA-HNA.

			tetrahydrocortisol; tetrahydro-11-deoxycorticosterone	
Mann-Whitney U Test	NA	NA	Free cortisol; free cortisone; 6β-hydroxycortisol; 18-hydroxycorticosterone; etio; androstenetriol; 16-oxo-androstendiol; pregnanediol; pregnanetriol; 11-oxo-pregnanetriol; pregnenediol; 5-pregnen,3α,16α,20α-triol; 5-Pregnen,3α,17α,20α-triol; 6-hydroxy-pregnanolone; THS; hexahydro-11-deoxycortisol; 21-deoxy-tetrahydrocortisol; tetrahydro-11-deoxycorticosterone; THE; THB; THF; a-THF; α + β-cortolones; α + β-cortols; hexahydrocorticosterone; dihydrocortisone; dihydrocortisol; cortisone; cortisol	The metabolites were significantly higher in ACC-CS compared to ACA-HNA.
Mann-Whitney U Test	NA	NA	Free cortisol; free cortisone; 6β-hydroxycortisol; etio; androstenetriol; androstendiol-17β; DHEA; 16-hydroxy-DHEA; 11-hydroxy-etiocholanolone; androstenetriol; 16-oxo-androstendiol; 17-hydroxy-pregnanolone; pregnanediol; pregnanetriol; 11-oxo-pregnanetriol; pregnenediol; 5-pregnen,3α,16α,20α-triol; 5-pregnen,3α,17α,20α-triol; 6-hydroxy-pregnanolone; THS; hexahydro-11-deoxycortisol; 21-deoxy-tetrahydrocortisol; tetrahydro-11-deoxycorticosterone; THE; THB; THF; α + β-Cortolones; α + β-cortols; cortisol	The metabolites were significantly higher in ACC-CS compared to ACA-CS.
Mann-Whitney U Test	NA	NA	(THE + THF + a-THF)/11-oxo-pregnanetriol; (THE + THF + a-THF)/THS	The ratio was lower in ACA-HNA, ACC-CS, ACC compared to the healthy group.
Mann-Whitney U Test	NA	NA	(THE + THF + a-THF)/pregnanetriol	The ratio is lower in ACC compared to ACA-HNA and the healthy group.
Mann-Whitney U Test	NA	NA	(THE + THF + a-THF)/THS	The ratio is lower in ACC, ACC-CS compared to ACA-HNA.

**Kerkhofs *et al.* (2015) [6]**	ROC analysis	NA	NA	THS; pregnanediol; pregnanetriol; etio; ADT; THF; THE; 11-KE; β-cortolone; α-cortolone; α-cortol; 11-HA; 11-HE; epi-pregnanolone	15 metabolites had a sensitivity > 0.9 for detecting ACC along with AUC range from 0.70 – 1.00 and specificity range from 0.02-0.99.
**Dai *et al.* (2014) [7]**	Binary logistic regression	NA	Urinary creatinine	Cortisol; androstanediol; a-THF; and epitestosterone	The model predicts the healthy state and liver disease (LC and early HCC) with an accuracy of 0.86 and 0. 94, respectively. In addition, AUC was 0.972, which exhibits better capability compared to AFP for discrimination.
	Binary logistic regression	NA	Urinary creatinine	a-THF and epitestosterone	The AUC was 0.938, and the accuracy for predicting LC and early HCC were 0.86 and 0.89, respectively.
**Perna *et al.* (2014) [8]**	Kruskal-Wallis nonparametric test	NA	NA	DHEA; 16α-hydroxy-DHEA; androstenetriol; 17β-androstenediol; c19 steroid sulfates; pregnenediol; pregnenetriol; 17HP; pregnanediol	These metabolites are significantly lower in ACA compared to ACC.
	Kruskal-Wallis nonparametric test	NA	NA	Cortisol metabolites/C19 steroid sulfates	The ratio is significantly higher in ACA compared to ACC.
**Konieczna *et al.* (2013) [9]**	Kolmogorov-Smirnov; Wald-Wolfowitz and Mann-Whitney U tests	NA	NA	Cortisone; cortisol; epitestosterone; progesterone	Significantly higher in cancer patients versus healthy, and in male bladder cancer versus male healthy. Progesterone level is statistically significant in all kinds of cancers. Testosterone and epitestosterone level also shows significant differences between groups.
	Kolmogorov-Smirnov; Wald-Wolfowitz and Mann-Whitney U tests	NA	NA	Cortisol; cortisone	Significantly higher in bladder cancer patients versus healthy and male cancer versus male healthy.
	Kolmogorov-Smirnov; Wald-	NA	NA	Progesterone	Significantly lower in all type of cancers (bladder, kidney, prostate, testis, and others) compared to the healthy patient in both genders.

	Wolfowitz and Mann-Whitney U tests				
	Kolmogorov-Smirnov; Wald-Wolfowitz and Mann-Whitney U tests	NA	NA	Epitestosterone	Significantly higher in female cancer patients versus female healthy, female kidney cancer versus female healthy, and prostate cancer patient versus healthy. Significantly lower in bladder cancer versus healthy patient, male cancer patient versus male healthy, and female bladder cancer patient versus female healthy.
	Kolmogorov-Smirnov; Wald-Wolfowitz and Mann-Whitney U tests	NA	NA	Testosterone	Significantly higher in female cancer patients versus female healthy, and female kidney cancer versus female healthy. Significantly lower in female bladder cancer versus female healthy.
	PLS-DA	NA	NA	Cortisol; cortisone; progesterone; epitestosterone; testosterone; corticosterone	The sensitivity of 0.81 and specificity of 0.74 when comparing healthy vs. cancer patient.
**Konieczna *et al.* (2013) [10]**	ANOVA test	NA	Urinary creatinine	Cortisone	Significantly higher in cancer patients versus healthy subjects.
	PLS-DA	NA	Urinary creatinine	Cortisol; cortisone; corticosterone; testosterone; epitestosterone; progesterone	Model-predicted cancer and healthy groups with a sensitivity of 1.00 and specificity of 0.89.
**Arlt *et al.* (2011) [11]**	Generalized matrix relevance learning vector quantization	NA	By subtracting the relevant means obtained in healthy group and dividing by the corresponding standard deviation	THS; pregnenediol; and pregnenetriol	The three most important steroids in differentiating ACC and ACA with AUC, sensitivity, and specificity of model were 0.94, 0.87, and 0.87.

	Generalized matrix relevance learning vector quantization	NA	By subtracting the relevant means obtained in healthy group and dividing by the corresponding standard deviation	Etio; pregnenetriol; pregnenediol; 5α-THA; THS; pregnanediol; pregnanetriol; tetrahydro-11-deoxycortisol	The nine most important steroids in differentiating ACC and ACA with AUC, sensitivity, and specificity of model were 0.96, 0.88, and 0.88, respectively.
	Generalized matrix relevance learning vector quantization	NA	By subtracting the relevant means obtained in healthy group and dividing by the corresponding standard deviation	32 metabolite[1]	Model of all 32 metabolites differentiates ACC and ACA with AUC, sensitivity, and specificity of model were 0.97, 0.90, and 0.90, respectively.
**Bufa *et al.* (2010) [12]**	Mann-Whitney U Test	NA	Urinary creatinine	ADT; Etio; 11-OH-ADT; 11-OH-Etio; pregnanetriol; D5-PT; THE; THA; THB; a-THB; THF; aTHF; α-cortolone; β-cortolone; α-cortol	Significantly lower in adenocarcinoma endometrii group than the control group.
	Mann-Whitney U Test	NA	BMI	ADT; Etio; 11-OH-ADT; 11-OH-Etio; PT; THE; THF; α-cortolone; β-cortolone	Significantly lower in adenocarcinoma endometrii group than the control group.
**Bufa *et al.* (2008) [13]**	Mann-Whitney U Test	NA	Urinary creatinine level	DHEA; 16-OHD; pregnanediol; pregnanetriol; androstenetriol; THA; THB; a-THB; a-THF	Significantly higher in the postmenopausal women with epithelial ovarian cancer than in the controls.
**Drafta *et al.* (1982) [14]**	Student t-test	NA	NA	Testosterone; testosterone/DTH; estrone; estradiol; estrone/testosterone; estradiol/testosterone; estrone+ estradiol/testosterone; cortisol	Testosterone, testosterone /DTH are significantly higher in the cancer group compared to benign or control group. Estrone, estradiol is significantly higher in the control group compared to the benign and cancer group. estrone/testosterone, estradiol/testosterone, estrone+ estradiol/testosterone significantly higher in control or benign group compared to cancer group. Cortisol is significantly higher in the benign group compared to the control or cancer group and significantly higher in the cancer group compared to the control group.
**Trabert *et al.* (2019) [15]**	Conditional logistic regression	Matching factors[1]	NA	Androsterone-glucuronide	Androsterone-glucuronide has significantly increased the risk of non-serous ovarian cancer when compare tertile 3 and tertile 1 (OR: 4.36, 95% CI: 1.68 to 11.32).

**Petrick *et al.* (2018) [16]**	Logistic regression	Age, BMI, at interview, education, gastroesophageal reflux disease symptoms, H. pylori seropositivity, smoking status	NA	DHEA; 5-diol; testosterone; DHT; ADT; estrone; estradiol; parent estrogens; free testosterone; free DHT; free estradiol	A higher level of individual steroid associated with significantly decreased odds of esophageal adenocarcinoma.
	Logistic regression	Age, BMI, at interview, education, gastroesophageal reflux disease symptoms, H. pylori seropositivity, smoking status	NA	Testosterone/parent estrogens ratio; testosterone/estradiol ratio	Comparing quartile 4 versus 1, testosterone: parent estrogens ratio and testosterone: estradiol ratio correlated with 1.5- and 2.6-times the odds of esophageal adenocarcinoma.
**Petrick *et al.* (2018) [17]**	Logistic regression	Age, education, smoking status, BMI, and diabetes	NA	DHEA; estradiol; free estradiol	Doubling in DHEA, estradiol, free estradiol was associated with reduced risk of esophageal and gastric cardia adenocarcinoma by 38% (OR=0.62, 95% CI: 0.47 to 0.82), 34% (OR=66, 95% CI: 0.45 to 0.98), and 35% (OR=0.65, 95% CI: 0.43 to 0.99).
**Sampson *et al.* (2017) [18]**	Logistic Regression	Confounding factors[1]	NA	Total estrogen level (15 estrogens/estrogen metabolites)	The total level significantly increased the risk of breast cancer (OR: 1.48, 95% CI: 1.20 to 1.82) when comparing 90th to 10th percentiles.
	Logistic Regression	Confounding factors[1]	NA	Estrone; estradiol; 2-hydroxyestrone; 2-hydroxyestradiol; 2-methoxyestrone; 2-hydroxyestrone-3-methyl ether; 4-hydroxyestrone; 16α-hydroxyestrone; estriol; 16-ketoestradiol; 16-epiestriol; 17-epiestriol	These estrogens/estrogen metabolites significantly increased the risk of breast cancer with OR range from 1.34 to 1.88 when comparing the 5th quintile to 1st quintile.
	Logistic Regression	Confounding factors[1]	NA	2-hydroxylation pathway metabolites/total metabolites; and 2-hydroxylation/16-hydroxylation pathway metabolites ratio	The ratios significantly reduced the risk of breast cancer (OR: 0.61, 95% CI: 0.46 to 0.80 and OR:0.66, 95% CI:0.52 to 0.84, respectively) when comparing 90th to 10th percentiles.

**Brinton and Trabert *et al.* (2016) [19]**	Logistic Regression	Confounding factors[1]	NA	Unconjugated estradiol; estrone	Unconjugated estradiol and estrone significantly increased the risk of endometrial cancers (OR: 6.19, 95% CI: 2.95 to 13.03, and OR: 3.19, 95% CI: 1.69 to 6.04).
	Logistic Regression	Confounding factors[1] and estradiol level	NA	2-hydroxyestrone; 2-hydroxyestradiol; 2-methoxyestrone; 2-methoxyestradiol; 2-hydroxyestrone-3-methyl ether; 4-hydroxyestrone; 4-methoxyestrone; 4-methoxyestradiol; 16α-hydroxyestrone; estriol; 16-ketoestradiol; 16-epiestriol; 17-epiestriol	Estrogens metabolites significantly increased the risk of endometrial cancer when comparing the highest quintile (quintile 5th) and lowest quintile (quintile 1st) with OR range from 2.1 to 4.0.
**Moore *et al.* (2016) [20]**	Logistic Regression	Confounding factors[1]	Creatinine	Estrone; estradiol; 2-hydroxyestrone; 2-methoxyestrone; 2-hydroxyestrone-3-methyl ether; 4-hydroxyestrone; estriol; 16-ketoestradiol; 16-epiestriol; 16α-hydroxyestrone; and 17-epiestriol	Estrogen metabolites significantly increased the risk of breast cancer with OR range from 1.24 to 1.56.
**Trabert *et al.* (2016) [21]**	Logistic Regression	Confounding factors[1]	NA	2-methoxyestrone	2-methoxyestrone significantly increased risk of ovarian cancer (OR: 2.03, 95% CI: 1.06 to 3.88) when comparing quintile 5 and quintile 1.
	Logistic Regression	Confounding factors[1]	NA	Estrone; unconjugated estradiol; 2-hydroxyestrone; unconjugated 2-methoxyestrone; conjugated 2-methoxyestrone; unconjugated 2-methoxyestradiol; 4-hydroxyestrone; 4-methoxyestrone; 4-methoxyestradiol; 16α-hydroxyestrone; conjugated estriol; 16-ketoestradiol; 16-epiestriol	Estrogens and estrogen metabolites were significantly associated with non-serous ovarian cancer when comparing the highest quintile and lowest quintile with OR range from 2.55 to 4.27.
**Dallal *et al.* (2016) [22]**	Cox regression	Study design variables[1], clinic, trial participation status, BMI adjustment	NA	Estradiol	Higher estradiol level significantly increased the risk of endometrial cancer ($HR_{tertile3vstertile1}$ = 4.38, 95% CI: 1.82 to 10.5).
	Cox regression	Study design variables[1], clinic, trial participation	NA	2-hydroxyestrone; 4-hydroxyestrone	For the 2-pathway and 4-pathway, the two steroid levels significantly increased risk of endometrial cancer when comparing the highest tertile to the lowest (2-hydroxyestrone,

		status, BMI adjustment			HR=3.24, 95% CI: 1.38 to 7.59; 4-hydroxyestrone: HR=2.83, 95% CI 1.26 to 6.36)
	Cox regression	Study design variables[1], clinic, trial participation status, BMI adjustment		16α-hydroxyestrone; 17-epiestriol	For 16-pathway, 16α-hydroxyestrone significantly increased the risk of endometrial cancer (HR:2.37, 95% CI: 1.05 to 5.35) while 17-epiestriol reduce the risk of endometrial cancer (HR=0.37, 95% CI: 0.16 to 0.88) when comparing the highest tertile to lowest tertile.
**Schairer *et al.* (2015) [23]**	Cox proportional hazards model	Confounding factors[1]	NA	unconjugated estradiol	Unconjugated estradiol significantly increased the risk of breast cancer (HR: 1.66, 95% CI: 1.00 to 2.76) and was higher in estrogen receptor-positive breast cancer (HR: 2.06, 95% CI: 1.14 to 3.71)
**Black *et al.* (2014) [24]**	Logistic Regression	Age at blood draw, BMI and SHBG	NA	2-methoxyestrone; 4-methoxyestrone;	The estrogen metabolites significantly decreased the risk of prostate cancer with (OR: 0.53, 95 CI: 0.29 to 0.94 and OR: 0.45, 95% CI: 0.22 to 0.92, respectively) when comparing the 4th quartile with 1st quartile.
	Logistic Regression	Age at blood draw, BMI and SHBG	NA	2-hydroxyestrone:16α -hydroxyestrone	The ratio significantly increased the risk of prostate cancer (OR: 2.44, 95% CI: 1.34 to 4.45) when comparing the 4th quartile with 1st quartile.
**Falk *et al.* (2013) [25]**	Logistic Regression	Confounding factors[1]	NA	Estradiol; conjugated estrone; unconjugated 2-methoxyestradiol; and estriol	These steroids significantly increased the risk of breast cancer when comparing the 3rd quartile and 1st quartile with OR of 2.03 (95%CI: 1.15 to 3.57), 1.86 (95% CI: 1.06 to 3.24), 1.89 (95% CI: 1.09 to 3.29) and 1.85 (95% CI: 1.04 to 3.29), respectively.
	Logistic Regression	Confounding factors[1]	NA	16-epiestriol	16-epiestriol significantly increased the risk of breast cancer when comparing 2nd, 3rd, 4th quartile with the 1st quartile with OR of 2.06 (95% CI: 1.16 to 3.66), 1.81 (95% CI: 1.01 to 3.25), and 1.95 (95% CI: 1.08 to 3.52).
	Logistic Regression	Confounding factors[1]	NA	2-hydroxyestrone, 3-methyl ether; conjugated 2-methoxyestradiol	The steroids were inversely related to the increasing risk of breast cancer with OR of 0.57 (95% CI: 0.33 to 0.99) and 0.46 (95% CI: 0.25 to 0.84).
**Dallal *et al.* (2013) [26]**	Cox	Geographical clinic site and trial	NA	Estradiol; 2-methoxyestrone; estriol; 17-epiestriol; 16-ketoestradiol; 16-epiestriol	Estradiol, 2-Methoxyestrone, and 16-pathway metabolites significantly increased the risk of breast cancer with HR range from 1.46 to 1.98 when comparing quintile 5 and quintile 1.

	proportional hazards model	participation status			
	Cox proportional hazards model	Geographical clinic site and trial participation status	NA	2-hydroxylation pathway/parent estrogen and 4-hydroxylation pathway/ parent estrogen	The increasing of ratio of the 2-hydroxylation pathway (HR:0.69, 95% CI: 0.46 to 1.05) and 4-hydroxylation pathway (HR:0.61, 95% CI: 0.40 to 0.93) to parent estrogens (estradiol and estrone) significantly reduced the risk of breast cancer when comparing quintile 5 and quintile 1.
**Fuhrman *et al.* (2012) [27]**	Cox regression	Study factors[2]	NA	Unconjugated estradiol	Unconjugated estradiol associated with the risk of breast cancer (HR = 2.07, 95% CI: 1.19 to 3.62).
**Audet-Walsh *et al.* (2010) [28]**	Logistic regression	Age, BMI	NA	DHEA; DHEA-S; 5-Diol; 4-Dione; testosterone, DHT; ADT-G; 3α-Diol-17G; estrone; estradiol; E_1-S; estrone glucuronide; 2-methoxy-estrone-3-glucuronide; 2-methoxyestradiol-3-glucuronide	The elevated level of steroids is significant increased the risk of endometrial cancer with OR range from 1.12 to 13.27.<br>In endometrial cancer patient, elevated endogenous estrogens are likely to have low-grade and less invasive cancers.<br>Estrogens and their metabolites were much strongly correlated in endometrial cancer cases than in healthy postmenopausal women<br>Estrogens were predictive of type I endometrioid cancers, low-grade, and noninvasive tumors.
**Yang *et al.* (2009) [29]**	Mann-Whitney U Test	NA	NA	4-OHE1-1-N7Gua[2]; 4-OHE1-1-N3Ade[3]; 2-OHE1-6-N3Ade[3]	The levels of 4-OHE1(E2)-1-N7Gua, 4-OHE1-(E2)-1-N3Ade, and 2-OHE1(E2)-6-N3Ade are significantly higher in PC group than the control group and could be considered as potential biomarkers for PC risk prediction.
**Lévesque *et al.* (2019) [30]**	Multivariable Cox model	Age, PSA, Gleason scores, pathological T stage, margin, and nodal status	NA	5-diol; DHEA-S	In high-risk PC patient, increment in log-transformed androstenediol and DHEA-S levels were significantly associated with disease-free survival with HR of 1.47 and 1.24, respectively.<br>Log-transformed androstenediol level associated with disease-free-survival (HR: 1.19, 95% CI: 1.03 to 1.37) in PC patient.<br>Log-transformed androstenediol and DHEA-S levels increased the risk of recurrence between PC quartile 1 and quartile 4 (both Q4/Q1: HR = 1.34, 95% CI: 1.06 to 1.70).

	Multivariable Cox model	Age, PSA, Gleason scores, pathological T stage, margin, and nodal status	NA	5-diol; DHEA; DHEA-S; 3α-diol-17G	In high-risk PC patient, the untransformed steroid hormone levels were significantly linked to disease-free-survival with HR of 1.17, 1.16, 1.14, and 1.17, respectively.
**Audet-Delage *et al.* (2018) [31]**	Cox regression	NA	NA	Estriol	Comparing preoperative estriol level, those with estriol levels below median (<= 30.5 pg/ml) have higher risk of recurrence (HR=0.27, 95% CI: 0.09 to 0.80) and lower overall survival (log rank p-value=0.002) after surgery compared to those with higher median estriol levels (> 30.5 pg/ml).
	Cox regression	NA	NA	E1-S; 4-methoxyestradiol	Elevated E1-S levels were associated with a higher risk of recurrence (HR: 2.67, 95% CI: 1.02 to 6.99).<br>Elevated postoperative levels of 4-methoxyestradiol reduced the possibility to experience recurrence (HR=0.34, 95% CI: 0.07 to 0.89) after adjustment of prognostic factors.
	Wilcoxon signed rank test for paired data	OC, HRT, age, BMI	NA	DHEA; DHEA-S; 5-diol; 4-dione; testosterone; DHT; ADT; ADT-G; androstane-3α; 17β-diol 3-glucuronide; 3α-diol-17G; E_1-S; estrone; estradiol; 4-methoxyestrone; 4-methoxyestradiol	Significantly decreased after surgery for one month compared to preoperative condition except for 4-methoxyestradiol.
	Wilcoxon signed rank test for paired data	Age, BMI	NA	DHEA-S; DHEA; 5-diol; ADT-G; androstane-3α, 17β-diol 3-glucuronide	Significantly increased in postoperative women compared to the healthy group.
**Plenis *et al.* (2013) [32]**	Separate-variances t-test; Mann-Withney U test	NA	NA	Cortisone; cortisol; epitestosterone	Significantly different in the healthy versus the NET patient.
	Student's t-test; Mann-Withney U test	NA	NA	Cortisone; cortisol	Significantly different in healthy male versus NET male patients.

	Separate-variances t-test	NA	NA	Cortisone; cortisol	Significantly different in healthy female versus NET female patients.
	Separate-variances t-test; Mann-Withney U test	NA	NA	Cortisone; cortisol; testosterone; progesterone	Significantly different in healthy female versus NET male patients.
	Student's t-test; Separate-variances t-test; Mann-Withney U test	NA	NA	Cortisone; cortisol; testosterone; epitestosterone; progesterone	Significantly different in healthy male versus NET patient female.
**Lévesque *et al.* (2013) [33]**	NA	Age, smoking status	NA	DHEA; ADT	SRD5A1 rs166050, the increased risk of PC, was associated with higher levels of DHEA, ADT.
	NA	Age, smoking status	NA	DHT and androstane-3β -17β -diol	The level of the steroids was affected by SRD5A2 polymorphism (rs676033).
	NA	Age, smoking status	NA	Testosterone	Carriers of SRD5A rs2208532 SNP have a significantly higher level.
	NA	Age, smoking status	NA	3α-diol-17G	rs12470143A, the protective marker, was associated significantly with a higher level of androstane-3α, 17β-diol-17 glucuronide. In particular, rs2208532G was associated with a lower concentration of androstane-3α, 17β-diol-17 glucuronide levels.
**Thomas *et al.* (1982) [34]**	Log-rank test	NA	NA	ADT; aetiocholanolone	Patients who have urinary ADT, aetiocholanolone level lower than median value display a significantly higher breast cancer recurrence rate after mastectomy.
**Zang *et al.* (2014) [35]**	SVM	NA	NA	3β;16α-dihydroxyandrostenone sulfate; cortolone-3-glucuronide; pregnanetriol glucuronide; 4-dione; ADT-sulfate; 5α-dihydrotestosterone sulfate; etiocholanolone sulfate	Included in panel A[4] which has 40 discriminant features.

	SVM	NA	NA	3β;16α-dihydroxyandrostenone sulfate; cortolone-3-glucuronide; pregnanetriol glucuronide; 4-dione	Included in panel B[5], C[6], D[7], E[8] which have 38, 35, 28, 25 discriminant features, respectively.
	SVM	NA	NA	Cortolone-3-glucuronide; pregnanetriol glucuronide; 4-dione	Included in panel F[9] and G[10] which have 22 and 17 discriminant features, respectively.
**Song *et al.* (2012) [36]**	Nonparametric Wilcoxon test	NA	Sum of the peak intensities in the sample (total peak area)	Cholesta-3,5-diene; cholest-5-en-3-ol	The metabolite levels significantly lower compared to control groups.<br>Cholesta-3,5-diene and cholest-5-en-3-ol (VIP > 1) are the greatest contributors to differentiating between the two groups.
**Moore *et al.* (2018) [37]**	Conditional logistic regression	Multiple factors[1]	NA	16α-hydroxy DHEA 3-sulfate	The BMI-associated steroid independently increased the risk of invasive breast cancer and estrogen receptor–positive (ER+) breast cancer (OR: 1.65, 95% CI 1.22 2.22 and OR: 1.84, 95% CI 1.27 2.67, respectively).
	Conditional logistic regression	Multiple factors[1]	NA	4-androsten-3beta,17beta-diol	The BMI-associated steroid significantly increased the risk of breast cancer and estrogen receptor–positive (ER+) breast cancer (OR: 1.50, 95% CI: 1.11 to 2.02 and OR: 2.11, 95% CI: 1.44 to 3.09, respectively) when comparing the 90th and 10th percentile.
**Huang *et al.* (2017) [38]**	t-test	Age	The batch median followed by log-transformation	5α-androstan-3α,17α-diol disulfate	Significantly lower in men in T3 (stage 3) compared to controls.
	t-test	NA	NA	E1-S; 5α-pregnan-3β, 20α-diol disulfate; 7α-hydroxy-3-oxo-4-cholestenoate; 4-androsten-3α, 17α-diol monosulfate	Significantly higher level in men in T4 (stage 4) compared to controls.
**Mondul *et al.* (2015) [39]**	Conditional logistic regression	Matching factors[1]	Median value	16α-hydroxy DHEA 3-sulfate	The steroids significantly reduced the risk of prostate cancer (OR: 0.81, 95% CI: 0.66 to 1.00).
**Huang *et al.* (2018) [40]**	Cox regression	Age at diagnosis, cancer stage at diagnosis, and year of diagnosis	Batch median value	4-androsten-3β,17β-diol disulfate; pregnenolone sulfate; 5α -androstan-3β,17β-diol disulfate; 5α-androstan-3α,17α diol monosulfate; pregnen-diol disulfate	The top five sterols/steroid metabolites associated with the mortality of prostate cancer (high vs. low tertile HRs of 2.9, 2.5, 1.9, 2.2 and 1.8, respectively).

	Kaplan-Meier survival plots	NA	NA	44 metabolites[1]	The increase in sterol/steroid metabolites are linked to worse prostate cancer-specific mortality, particularly among cases diagnosed at least 7 years after blood collection (Log-rank p-value = 0.0021).
**Ye *et al.* (2014) [41]**	Correlation analysis	NA	NA	β-sitosterol	Level of β-sitosterol was increased in both S and NS patients following chemotherapy.
**Zhou *et al.* (2014) [42]**	Student's t-test	NA	NA	Tetrahydroaldosterone-3-glucuronide; hydroxyandrosterone-3-glucuronide; DHEA-S	Significantly lower in early recurrence HCC stage compare to lately HCC.
	PLS-DA	NA	NA	Cholestane-tetrol glucuronide; cholesterol glucuronide; cholesterol sulfate	Significantly higher in early recurrence HCC stage compare to lately HCC. Cholesterol sulfate was one of three important features in the model used for predicting the high risk of early recurrence of HCC in two years. The AUC of the model is 0.88 in the test set.
**Miller *et al.* (2015) [43]**	Paired two sample t-tests	NA	NA	ADT-sulfate; epiandosterone-sulfate; pregnendiol disulfate; 4-androsten-3β,17β diol disulfate 2	The significantly reduced of ADT-sulfate, epiandrosterone sulfate, 4-androsten-3β,17β-diol disulfate 2, and pregnendiol disulfate were observed in the group having the limonene intervention.
**Ghataore *et al.* (2012) [44]**	One-way ANOVA with Bonferroni post hoc	NA	NA	5α/5β-tetrahydrocortisol	Significant decrease in hydrocortisone, hydrocortisone + mitotane group, 5ARD2 deficiency group, finasteride, dutasteride group compared to the normal group.
**Saylor *et al.* (2012) [45]**	Paired t-test	NA	Median of the values (block normalization)	DHEA-S; epiandrosterone sulfate; ADT-sulfate; cortisol; 4-androsten- 3β,17β -diol disulfate 1; 4-androsten-3β,17β-diol disulfate 2; 5α-androstan-3β,17β-diol disulfate; pregnen-diol disulfate; pregn steroid monosulfate; andro steroid monosulfate 1	The metabolites significantly decreased after treatment with androgen deprivation therapy in men with PC.

1: More detail in the reference

2: Age (study entry, the birth of the first child, menarche, natural menopause); blood collection time; type of menopause; family history of breast cancer; the individual history of benign breast disease; hormone therapy.

3: The 2- and 4-catechol estrogens depurinating DNA adducts

4: This panel has 93.0; 94.3 and 92.1 in accuracy; specificity; and sensitivity; respectively.

5: This panel has 91.2; 90.6 and 91. 7 in accuracy; specificity; and sensitivity; respectively.

6: This panel has 90.2; 87.2; and 91.8 in accuracy; specificity; and sensitivity; respectively.

7: This panel has 86.1; 87.2; and 85.3 in accuracy; specificity; and sensitivity; respectively.

8: This panel has 84.4; 80.0 and 88.8 in accuracy; specificity; and sensitivity; respectively.

9: This panel has 85.0; 80.0; and 88.8in accuracy; specificity; and sensitivity; respectively.

10: This panel has 80.0; 81.0 and 79.3 in accuracy; specificity; and sensitivity; respectively.

AFP: Alpha-fetoprotein; AUC: Area under the curve; ACC: Adrenocortical carcinoma; ACA: Adrenocortical adenoma; ADT: Androgen deprivation therapy; AIC: Akaike Information Criteria; ACA-HNA: Adrenocortical adenoma hormonally non-active adenomas; ACA-CS: Adrenocortical adenoma with Cushing's syndrome; LC: liver cancer; NET: Neuroendocrine Tumor; NFAA: Nonfunctioning adrenal adenoma; NA: Not available; PC: prostate cancer; PCC/PGL: phaeochromocytoma/paraganglioma; SD: standard deviation, SVM: Support-vector machine

ADT: Androsterone; ADT-G: ADT-glucuronide; E1-S: estrone-sulfate; 16-OHD: 16-Hydroxy-DHEA; THA: Tetrahydro-11-dehydrocorticosterone; THB: Tetrahydro-corticosterone; a-THB: Allo-tetrahydro-corticosterone; THF: tetrahydrocortisol; a-THF: Allo-tetrahydrocortisol; THS: tetrahydrodeoxycortisol; THE: tetrahydrocortisone; 5α-THA: 5α-tetrahydro-11- dehydrocorticosterone; DHA: 3 Dehydroepiandrosterone; DHEA: Dehydroepiandrosterone; DHEA-S: dehydroepiandrosterone sulfate; DHT: dihydrotestosterone; 17HP: 17α-hydroxypregnenolone; Etio: etiocholanolone; 5PT: 5-pregnenetriol; 5PD: 5-pregnenediol; 11-KE: 11-keto-etiocholanolone; 11-HA: 11-hydroxy-androsterone; 11-HE: 11-hydroxy-eticholanolone; 5-diol: androstenediol; 4-dione: androstenedione; 3α-diol-17G: androstane-3α; 17β-diol 17-glucuronide; 4MeO-E2: 4-methoxyestradiol; 11-OH-Etio: 11β-hydroxy-etiocholanolone; 11-OH-ADT: 11β-hydroxyandrosterone

Reference

1. Schweitzer, S.; Kunz, M.; Kurlbaum, M.; Vey, J.; Kendl, S.; Deutschbein, T.; Hahner, S.; Fassnacht, M.; Dandekar, T.; Kroiss, M. Plasma steroid metabolome profiling for the diagnosis of adrenocortical carcinoma. *European journal of endocrinology* **2019**, *180*, 117-125, doi:10.1530/eje-18-0782.
2. Hines, J.M.; Bancos, I.; Bancos, C.; Singh, R.D.; Avula, A.V.; Young, W.F.; Grebe, S.K.; Singh, R.J. High-Resolution, Accurate-Mass (HRAM) Mass Spectrometry Urine Steroid Profiling in the Diagnosis of Adrenal Disorders. *Clinical chemistry* **2017**, *63*, 1824-1835, doi:10.1373/clinchem.2017.271106.
3. Taylor, D.R.; Ghataore, L.; Couchman, L.; Vincent, R.P.; Whitelaw, B.; Lewis, D.; Diaz-Cano, S.; Galata, G.; Schulte, K.M.; Aylwin, S., et al. A 13-Steroid Serum Panel Based on LC-MS/MS: Use in Detection of Adrenocortical Carcinoma. *Clinical chemistry* **2017**, *63*, 1836-1846, doi:10.1373/clinchem.2017.277624.
4. Qian, X.; Zhan, Q.; Lv, L.; Zhang, H.; Hong, Z.; Li, Y.; Xu, H.; Chai, Y.; Zhao, L.; Zhang, G. Steroid hormone profiles plus α-fetoprotein for diagnosing primary liver cancer by liquid chromatography tandem mass spectrometry. *Clinica chimica acta; international journal of clinical chemistry* **2016**, *457*, 92-98, doi:10.1016/j.cca.2016.03.022.
5. Velikanova, L.I.; Shafigullina, Z.R.; Lisitsin, A.A.; Vorokhobina, N.V.; Grigoryan, K.; Kukhianidze, E.A.; Strelnikova, E.G.; Krivokhizhina, N.S.; Krasnov, L.M.; Fedorov, E.A., et al. Different Types of Urinary Steroid Profiling Obtained by High-Performance Liquid Chromatography and Gas Chromatography-Mass Spectrometry in Patients with Adrenocortical Carcinoma. *Horm Cancer* **2016**, *7*, 327-335, doi:10.1007/s12672-016-0267-0.
6. Kerkhofs, T.M.; Kerstens, M.N.; Kema, I.P.; Willems, T.P.; Haak, H.R. Diagnostic Value of Urinary Steroid Profiling in the Evaluation of Adrenal Tumors. *Horm Cancer* **2015**, *6*, 168-175, doi:10.1007/s12672-015-0224-3.
7. Dai, W.; Yin, P.; Chen, P.; Kong, H.; Luo, P.; Xu, Z.; Lu, X.; Xu, G. Study of urinary steroid hormone disorders: difference between hepatocellular carcinoma in early stage and cirrhosis. *Analytical and bioanalytical chemistry* **2014**, *406*, 4325-4335, doi:10.1007/s00216-014-7843-3.
8. Perna, V.; Taylor, N.F.; Dworakowska, D.; Schulte, K.M.; Aylwin, S.; Al-Hashimi, F.; Diaz-Cano, S.J. Adrenocortical adenomas with regression and myelolipomatous changes: urinary steroid profiling supports a distinctive benign neoplasm. *Clinical endocrinology* **2014**, *81*, 343-349, doi:10.1111/cen.12458.
9. Konieczna, L.; Belka, M.; Baczek, T.; Ruszkowski, M.; Struck, W.; Markuszewski, M.; Kaliszan, R.; Markuszewski, M. Advanced assessment of the endogenous hormone level as a potential biomarker of the urogenital tract cancer. *Combinatorial chemistry & high throughput screening* **2013**, *16*, 463-472.
10. Konieczna, L.; Baczek, T.; Belka, M.; Fel, A.; Markuszewski, M.; Struck, W.; Markuszewski, M.; Kaliszan, R. Steroid profiles as potential biomarkers in patients with urogenital tract cancer for diagnostic investigations analyzed by liquid chromatography coupled to mass spectrometry. *J Pharm Biomed Anal* **2013**, *73*, 108-115, doi:10.1016/j.jpba.2012.03.016.
11. Arlt, W.; Biehl, M.; Taylor, A.E.; Hahner, S.; Libe, R.; Hughes, B.A.; Schneider, P.; Smith, D.J.; Stiekema, H.; Krone, N., et al. Urine steroid metabolomics as a biomarker

tool for detecting malignancy in adrenal tumors. *The Journal of clinical endocrinology and metabolism* **2011**, *96*, 3775-3784, doi:10.1210/jc.2011-1565.
12. Bufa, A.; Biro, I.; Poor, V.; Molnar, G.; Kovacs, K.A.; Felinger, A.; Jeges, S.; Kilar, F.; Gocze, P.M. Altered urinary profiles of endogenous steroids in postmenopausal women with adenocarcinoma endometrii. *Gynecological endocrinology : the official journal of the International Society of Gynecological Endocrinology* **2010**, *26*, 10-15, doi:10.3109/09513590903159581.
13. Bufa, A.; Poór, V.; Bálint, A.; Molnár, S.; Jeges, S.; Pótó, L.; Gőcze, P.; Kilár, F. Endogenous Urinary Steroids in Postmenopausal Women with Epithelial Ovarian Cancer. *Chromatographia* **2008**, *68*, 131-135, doi:10.1365/s10337-008-0710-7.
14. Drafta, D.; Proca, E.; Zamfir, V.; Schindler, A.E.; Neacsu, E.; Stroe, E. Plasma steroids in benign prostatic hypertrophy and carcinoma of the prostate. *Journal of steroid biochemistry* **1982**, *17*, 689-693.
15. Trabert, B.; Michels, K.A.; Anderson, G.L.; Brinton, L.A.; Falk, R.T.; Geczik, A.M.; Harris, H.R.; Pan, K.; Pfeiffer, R.M.; Qi, L., et al. Circulating androgens and postmenopausal ovarian cancer risk in the Women's Health Initiative Observational Study. **2019**, *0*, doi:10.1002/ijc.32157.
16. Petrick, J.L.; Falk, R.T.; Hyland, P.L.; Caron, P.; Pfeiffer, R.M.; Wood, S.N.; Dawsey, S.M.; Abnet, C.C.; Taylor, P.R.; Guillemette, C., et al. Association between circulating levels of sex steroid hormones and esophageal adenocarcinoma in the FINBAR Study. *PloS one* **2018**, *13*, e0190325-e0190325, doi:10.1371/journal.pone.0190325.
17. Petrick, J.L.; Hyland, P.L.; Caron, P.; Falk, R.T.; Pfeiffer, R.M.; Dawsey, S.M.; Abnet, C.C.; Taylor, P.R.; Weinstein, S.J.; Albanes, D., et al. Associations Between Prediagnostic Concentrations of Circulating Sex Steroid Hormones and Esophageal/Gastric Cardia Adenocarcinoma Among Men. *Journal of the National Cancer Institute* **2019**, *111*, 34-41, doi:10.1093/jnci/djy082.
18. Sampson, J.N.; Falk, R.T.; Schairer, C.; Moore, S.C.; Fuhrman, B.J.; Dallal, C.M.; Bauer, D.C.; Dorgan, J.F.; Shu, X.O.; Zheng, W., et al. Association of Estrogen Metabolism with Breast Cancer Risk in Different Cohorts of Postmenopausal Women. *Cancer research* **2017**, *77*, 918-925, doi:10.1158/0008-5472.Can-16-1717.
19. Brinton, L.A.; Trabert, B.; Anderson, G.L.; Falk, R.T.; Felix, A.S.; Fuhrman, B.J.; Gass, M.L.; Kuller, L.H.; Pfeiffer, R.M.; Rohan, T.E., et al. Serum Estrogens and Estrogen Metabolites and Endometrial Cancer Risk among Postmenopausal Women. *Cancer epidemiology, biomarkers & prevention : a publication of the American Association for Cancer Research, cosponsored by the American Society of Preventive Oncology* **2016**, *25*, 1081-1089, doi:10.1158/1055-9965.Epi-16-0225.
20. Moore, S.C.; Matthews, C.E.; Ou Shu, X.; Yu, K.; Gail, M.H.; Xu, X.; Ji, B.T.; Chow, W.H.; Cai, Q.; Li, H., et al. Endogenous Estrogens, Estrogen Metabolites, and Breast Cancer Risk in Postmenopausal Chinese Women. *Journal of the National Cancer Institute* **2016**, *108*, doi:10.1093/jnci/djw103.
21. Trabert, B.; Brinton, L.A.; Anderson, G.L.; Pfeiffer, R.M.; Falk, R.T.; Strickler, H.D.; Sliesoraitis, S.; Kuller, L.H.; Gass, M.L.; Fuhrman, B.J., et al. Circulating Estrogens and Postmenopausal Ovarian Cancer Risk in the Women's Health Initiative Observational Study. *Cancer epidemiology, biomarkers & prevention : a publication of the American Association for Cancer Research, cosponsored by the American Society of Preventive Oncology* **2016**, *25*, 648-656, doi:10.1158/1055-9965.Epi-15-1272-t.

22. Dallal, C.M.; Lacey, J.V., Jr.; Pfeiffer, R.M.; Bauer, D.C.; Falk, R.T.; Buist, D.S.; Cauley, J.A.; Hue, T.F.; LaCroix, A.Z.; Tice, J.A., et al. Estrogen Metabolism and Risk of Postmenopausal Endometrial and Ovarian Cancer: the B approximately FIT Cohort. *Horm Cancer* **2016**, *7*, 49-64, doi:10.1007/s12672-015-0237-y.
23. Schairer, C.; Fuhrman, B.J.; Boyd-Morin, J.; Genkinger, J.M.; Gail, M.H.; Hoover, R.N.; Ziegler, R.G. Quantifying the Role of Circulating Unconjugated Estradiol in Mediating the Body Mass Index-Breast Cancer Association. *Cancer epidemiology, biomarkers & prevention : a publication of the American Association for Cancer Research, cosponsored by the American Society of Preventive Oncology* **2016**, *25*, 105-113, doi:10.1158/1055-9965.Epi-15-0687.
24. Black, A.; Pinsky, P.F.; Grubb, R.L., 3rd; Falk, R.T.; Hsing, A.W.; Chu, L.; Meyer, T.; Veenstra, T.D.; Xu, X.; Yu, K., et al. Sex steroid hormone metabolism in relation to risk of aggressive prostate cancer. *Cancer epidemiology, biomarkers & prevention : a publication of the American Association for Cancer Research, cosponsored by the American Society of Preventive Oncology* **2014**, *23*, 2374-2382, doi:10.1158/1055-9965.Epi-14-0700.
25. Falk, R.T.; Brinton, L.A.; Dorgan, J.F.; Fuhrman, B.J.; Veenstra, T.D.; Xu, X.; Gierach, G.L. Relationship of serum estrogens and estrogen metabolites to postmenopausal breast cancer risk: a nested case-control study. *Breast cancer research : BCR* **2013**, *15*, R34, doi:10.1186/bcr3416.
26. Dallal, C.M.; Tice, J.A.; Buist, D.S.M.; Bauer, D.C.; Lacey, J.V., Jr.; Cauley, J.A.; Hue, T.F.; Lacroix, A.; Falk, R.T.; Pfeiffer, R.M., et al. Estrogen metabolism and breast cancer risk among postmenopausal women: a case-cohort study within B~FIT. *Carcinogenesis* **2014**, *35*, 346-355, doi:10.1093/carcin/bgt367.
27. Fuhrman, B.J.; Schairer, C.; Gail, M.H.; Boyd-Morin, J.; Xu, X.; Sue, L.Y.; Buys, S.S.; Isaacs, C.; Keefer, L.K.; Veenstra, T.D., et al. Estrogen metabolism and risk of breast cancer in postmenopausal women. *Journal of the National Cancer Institute* **2012**, *104*, 326-339, doi:10.1093/jnci/djr531.
28. Audet-Walsh, E.; Lepine, J.; Gregoire, J.; Plante, M.; Caron, P.; Tetu, B.; Ayotte, P.; Brisson, J.; Villeneuve, L.; Belanger, A., et al. Profiling of endogenous estrogens, their precursors, and metabolites in endometrial cancer patients: association with risk and relationship to clinical characteristics. *The Journal of clinical endocrinology and metabolism* **2011**, *96*, E330-339, doi:10.1210/jc.2010-2050.
29. Yang, L.; Gaikwad, N.W.; Meza, J.; Cavalieri, E.L.; Muti, P.; Trock, B.; Rogan, E.G. Novel biomarkers for risk of prostate cancer: results from a case-control study. *The Prostate* **2009**, *69*, 41-48, doi:10.1002/pros.20850.
30. Levesque, E.; Caron, P.; Lacombe, L.; Turcotte, V.; Simonyan, D.; Fradet, Y.; Aprikian, A.; Saad, F.; Carmel, M.; Chevalier, S., et al. A Comprehensive Analysis of Steroid Hormones and Progression of Localized High-Risk Prostate Cancer. *Cancer Epidemiology Biomarkers & Prevention* **2019**, 10.1158/1055-9965.EPI-18-1002, cebp.1002.2018, doi:10.1158/1055-9965.EPI-18-1002.
31. Audet-Delage, Y.; Gregoire, J.; Caron, P.; Turcotte, V.; Plante, M.; Ayotte, P.; Simonyan, D.; Villeneuve, L.; Guillemette, C. Estradiol metabolites as biomarkers of endometrial cancer prognosis after surgery. *J Steroid Biochem Mol Biol* **2018**, *178*, 45-54, doi:10.1016/j.jsbmb.2017.10.021.
32. Plenis, A.; Miękus, N.; Olędzka, I.; Bączek, T.; Lewczuk, A.; Woźniak, Z.; Koszałka, P.; Seroczyńska, B.; Skokowski, J. Chemometric evaluation of urinary steroid

hormone levels as potential biomarkers of neuroendocrine tumors. *Molecules (Basel, Switzerland)* **2013**, *18*, 12857-12876, doi:10.3390/molecules181012857.

33. Lévesque, É.; Laverdière, I.; Lacombe, L.; Caron, P.; Rouleau, M.; Turcotte, V.; Têtu, B.; Fradet, Y.; Guillemette, C. Importance of 5α-Reductase Gene Polymorphisms on Circulating and Intraprostatic Androgens in Prostate Cancer. *Clinical Cancer Research* **2014**, *20*, 576-584, doi:10.1158/1078-0432.CCR-13-1100 %J Clinical Cancer Research.
34. Thomas, B.S.; Bulbrook, R.D.; Hayward, J.L.; Millis, R.R. Urinary androgen metabolites and recurrence rates in early breast cancer. *European journal of cancer & clinical oncology* **1982**, *18*, 447-451.
35. Zang, X.; Jones, C.M.; Long, T.Q.; Monge, M.E.; Zhou, M.; Walker, L.D.; Mezencev, R.; Gray, A.; McDonald, J.F.; Fernandez, F.M. Feasibility of detecting prostate cancer by ultraperformance liquid chromatography-mass spectrometry serum metabolomics. *J Proteome Res* **2014**, *13*, 3444-3454, doi:10.1021/pr500409q.
36. Song, H.; Peng, J.-S.; Dong-Sheng, Y.; Yang, Z.-L.; Liu, H.-L.; Zeng, Y.-K.; Shi, X.-P.; Lu, B.-Y. Serum metabolic profiling of human gastric cancer based on gas chromatography/mass spectrometry. *Brazilian journal of medical and biological research* **2011**, *45*, 78-85, doi:10.1590/S0100-879X2011007500158.
37. Moore, S.C.; Playdon, M.C.; Sampson, J.N.; Hoover, R.N.; Trabert, B.; Matthews, C.E.; Ziegler, R.G. A Metabolomics Analysis of Body Mass Index and Postmenopausal Breast Cancer Risk. *Journal of the National Cancer Institute* **2018**, *110*, 588-597, doi:10.1093/jnci/djx244.
38. Huang, J.; Mondul, A.M.; Weinstein, S.J.; Karoly, E.D.; Sampson, J.N.; Albanes, D. Prospective serum metabolomic profile of prostate cancer by size and extent of primary tumor. *Oncotarget* **2017**, *8*, 45190-45199, doi:10.18632/oncotarget.16775.
39. Mondul, A.M.; Moore, S.C.; Weinstein, S.J.; Karoly, E.D.; Sampson, J.N.; Albanes, D. Metabolomic analysis of prostate cancer risk in a prospective cohort: The alpha-tocolpherol, beta-carotene cancer prevention (ATBC) study. *International journal of cancer* **2015**, *137*, 2124-2132, doi:10.1002/ijc.29576.
40. Huang, J.; Weinstein, S.J.; Moore, S.C.; Derkach, A.; Hua, X.; Mondul, A.M.; Sampson, J.N.; Albanes, D. Pre-diagnostic Serum Metabolomic Profiling of Prostate Cancer Survival. *The journals of gerontology. Series A, Biological sciences and medical sciences* **2018**, 10.1093/gerona/gly128, doi:10.1093/gerona/gly128.
41. Ye, G.; Liu, Y.; Yin, P.; Zeng, Z.; Huang, Q.; Kong, H.; Lu, X.; Zhong, L.; Zhang, Z.; Xu, G. Study of induction chemotherapy efficacy in oral squamous cell carcinoma using pseudotargeted metabolomics. *J Proteome Res* **2014**, *13*, 1994-2004, doi:10.1021/pr4011298.
42. Zhou, L.; Liao, Y.; Yin, P.; Zeng, Z.; Li, J.; Lu, X.; Zheng, L.; Xu, G. Metabolic profiling study of early and late recurrence of hepatocellular carcinoma based on liquid chromatography-mass spectrometry. *J Chromatogr B Analyt Technol Biomed Life Sci* **2014**, *966*, 163-170, doi:10.1016/j.jchromb.2014.01.057.
43. Miller, J.A.; Pappan, K.; Thompson, P.A.; Want, E.J.; Siskos, A.P.; Keun, H.C.; Wulff, J.; Hu, C.; Lang, J.E.; Chow, H.H. Plasma metabolomic profiles of breast cancer patients after short-term limonene intervention. *Cancer prevention research (Philadelphia, Pa.)* **2015**, *8*, 86-93, doi:10.1158/1940-6207.Capr-14-0100.
44. Ghataore, L.; Chakraborti, I.; Aylwin, S.J.; Schulte, K.M.; Dworakowska, D.; Coskeran, P.; Taylor, N.F. Effects of mitotane treatment on human steroid

metabolism: implications for patient management. *Endocrine connections* **2012**, *1*, 37-47, doi:10.1530/EC-12-0028.

45. Saylor, P.J.; Karoly, E.D.; Smith, M.R. Prospective study of changes in the metabolomic profiles of men during their first three months of androgen deprivation therapy for prostate cancer. *Clinical cancer research : an official journal of the American Association for Cancer Research* **2012**, *18*, 3677-3685, doi:10.1158/1078-0432.CCR-11-3209.

Table S3. The QUADOMICS quality assessment results of the included studies.

Author	Year	Item 1	Item 2	Item 3	Item 4	Item 5	Item 6	Item 7	Item 8	Item 9	Item 10	Item 11	Item 12	Item 13	Item 14	Item 15	Item 16
**Schweitzer *et al.***	2018	Y	N/A	Y	Y	Y	Y	U	U	Y	Y	U	U	Y	N/A	Y	N
**Hines *et al.***	2017	Y	N/A	Y	N	Y	Y	Y	Y	Y	Y	Y	Y	Y	N/A	Y	N/A
**Taylor *et al.***	2017	N	N/A	Y	N	Y	Y	Y	Y	Y	Y	Y	U	Y	N/A	Y	N/A
**Qian *et al.***	2016	N	N/A	Y	N	Y	Y	Y	Y	Y	Y	Y	U	Y	N/A	Y	N
**Velikanova *et al.***	2016	N	N/A	Y	Y	N	N	Y	Y	Y	Y	Y	U	Y	N/A	Y	N/A
**Kerkhofs *et al.***	2015	N	N/A	Y	Y	N	U	Y	N	Y	N	Y	U	Y	N/A	Y	N
**Dai *et al.***	2014	N	N/A	Y	N	Y	Y	Y	Y	Y	Y	Y	U	Y	N/A	Y	Y
**Perna *et al.***	2014	Y	N/A	Y	Y	N	Y	Y	Y	Y	Y	Y	U	Y	N/A	Y	N/A
**Konieczna *et al.***	2013	N	N/A	Y	Y	N	U	Y	Y	Y	Y	Y	U	Y	N/A	Y	N
**Konieczna *et al.***	2013	Y	N/A	Y	N	Y	Y	U	U	Y	Y	U	U	Y	N/A	Y	Y
**Arlt *et al.***	2011	N	N/A	Y	Y	Y	Y	Y	Y	Y	Y	Y	U	Y	N/A	Y	N
**Bufa *et al.***	2009	N	N/A	Y	Y	N	Y	U	U	Y	Y	U	U	Y	N/A	Y	N/A
**Bufa *et al.***	2008	N	N/A	Y	N	Y	Y	U	U	Y	Y	U	U	Y	N/A	Y	N/A
**Drafta *et al.***	1981	N	N/A	Y	Y	N	Y	Y	Y	Y	N	Y	U	Y	N/A	Y	N/A
**Trabert *et al.***	2019	Y	N/A	Y	Y	N	Y	Y	Y	N	Y	U	U	Y	N/A	Y	N/A
**Petrick *et al.***	2018	Y	N/A	Y	Y	Y	Y	Y	Y	Y	Y	Y	N	Y	N/A	Y	N/A
**Petrick *et al.***	2018	Y	N/A	Y	Y	N	Y	U	U	Y	N	U	N	Y	N/A	Y	N/A
**Sampson *et al.***	2017	Y	N/A	Y	Y	Y	Y	Y	Y	Y	U	U	U	Y	N/A	Y	N/A
**Brinton and Trabert *et al.***	2016	Y	N/A	Y	Y	Y	Y	Y	Y	Y	N	Y	U	Y	N/A	Y	N/A
**Moore *et at.***	2016	Y	N/A	Y	Y	N	Y	Y	Y	Y	N	Y	U	Y	N/A	Y	N/A
**Trabert *et al.***	2016	Y	N/A	Y	Y	Y	Y	Y	Y	Y	Y	U	U	Y	N/A	Y	N/A
**Dallal *et al.***	2015	Y	N/A	Y	Y	Y	Y	U	U	Y	Y	U	U	Y	N/A	Y	N/A
**Schairer *et al.***	2015	Y	N/A	Y	Y	Y	Y	Y	Y	Y	Y	U	Y	Y	N/A	Y	N/A

**Black** *et al.*	2014	Y	N/A	Y	Y	Y	Y	Y	Y	Y	Y	Y	Y	Y	N/A	Y	N/A
**Falk** *et al.*	2013	Y	N/A	Y	Y	Y	Y	Y	Y	Y	Y	U	U	Y	N/A	Y	N/A
**Dallal** *et al.*	2013	Y	N/A	Y	Y	Y	Y	U	U	Y	Y	U	U	Y	N/A	Y	N/A
**Fuhrman** *et al.*	2011	Y	N/A	Y	Y	Y	Y	U	U	Y	Y	U	U	Y	N/A	Y	N/A
**Audet-Walsh** *et al.*	2011	Y	N/A	Y	Y	Y	Y	U	U	Y	Y	U	U	Y	N/A	Y	N/A
**Yang** *et al.*	2009	Y	N/A	Y	Y	Y	Y	U	U	Y	Y	U	Y	Y	N/A	Y	N/A
**Lévesque** *et al.*	2019	Y	N/A	Y	Y	Y	Y	Y	Y	Y	N	U	Y	Y	N/A	Y	N/A
**Audet-Delage** *et al.*	2018	Y	N/A	Y	Y	Y	Y	Y	Y	Y	Y	Y	U	Y	N/A	Y	N/A
**Plenis** *et al.*	2013	N	N/A	Y	N	Y	U	U	U	Y	Y	U	U	Y	N/A	Y	N/A
**Lévesque** *et al.*	2013	Y	N/A	Y	N	Y	Y	Y	Y	Y	Y	Y	U	Y	N/A	Y	N/A
**Thomas** *et al.*	1982	N	N/A	Y	N	N	Y	Y	Y	Y	N	Y	U	Y	N/A	Y	N/A
**Zang** *et al.*	2014	N	N/A	Y	Y	Y	Y	U	U	Y	Y	U	U	Y	N/A	Y	Y
**Song** *et al.*	2012	N	N/A	Y	Y	Y	Y	Y	Y	Y	Y	Y	Y	Y	N/A	Y	N
**Moore** *et at.*	2018	Y	N/A	Y	Y	Y	Y	Y	Y	Y	Y	U	U	Y	N/A	Y	N/A
**Haung** *et al.*	2017	N	N/A	Y	Y	Y	Y	U	U	Y	N	U	U	Y	N/A	Y	N/A
**Mondul** *et al.*	2015	Y	N/A	Y	Y	Y	Y	Y	Y	Y	Y	Y	U	Y	N/A	Y	N/A
**Huang** *et al.*	2018	Y	N/A	Y	Y	Y	Y	Y	Y	Y	Y	Y	Y	Y	N/A	Y	N/A
**Ye** *et al.*	2014	Y	N/A	Y	Y	Y	Y	Y	Y	Y	Y	Y	U	Y	N/A	Y	Y
**Zhou** *et al.*	2014	N	N/A	Y	Y	Y	Y	Y	Y	Y	Y	Y	U	Y	N/A	Y	Y
**Miller** *et al.*	2015	Y	N/A	Y	Y	Y	Y	Y	Y	Y	Y	Y	U	Y	N/A	Y	N/A
**Ghataore** *et al.*	2012	N	N/A	Y	Y	N	U	Y	Y	Y	Y	Y	U	Y	N/A	Y	N/A
**Saylor** *et al.*	2012	Y	N/A	Y	N	Y	Y	U	U	Y	Y	U	U	Y	N/A	Y	N/A

Item 1=Were selection criteria clearly described?

Item 2=Was the spectrum of patients representative of patients who will receive the test in practice?

Item 3= Was the type of sample fully described?

Item 4=Were the procedures and timing of biological sample collection with respect to clinical factors described with enough detail?

Item 5=Were handling and pre-analytical procedures reported in sufficient detail and similar for the whole sample? and, if differences in procedures were reported, was their effect on the results assessed?

Item 6= Is the time period between reference standard and index test short enough to be reasonably sure that the target condition did not change between the two tests?

Item 7=Is the reference standard likely to correctly classify the target condition?

Item 8=Did the whole sample or a random selection of the sample, receive verification using a reference standard?

Item 9=Did patients receive the same reference standard regardless of the index test result?

Item 10=Was the execution of the index test described in sufficient detail to permit replication of the test?

Item 11=Was the execution of the reference standard described in sufficient detail to permit its replication?

Item 12=Were the index test results interpreted without knowledge of the results of the reference standard?

Item 13= Were the reference standard results interpreted without knowledge of the results of the index test?

Item 14=Were the same clinical data available when test results were interpreted as would be available when the test is used in practice?

Item 15=Were uninterpretable/ intermediate test results reported?

Item 16=Is it likely that the presence of overfitting was avoided?

Y=criteria achieved, **N**=criteria not achieved, **U** =unclear or not stated, **N/A**=not applicable.

Table S4. The steroids reported in the included studies.

Steroid name	The number of studies reporting steroid	HMDB ID	ACC	PC	BC	BlC	EC	LC	KC	TC	NET	OC	OrC	E/GC	Total
Estradiol	15	HMDB0000151	✔	✔	✔		✔	✔				✔		✔	7
Dehydroepiandrosterone	11	HMDB0000077	✔	✔			✔	✔				✔		✔	6
Cortisol	10	HMDB0000063	✔	✔		✔		✔	✔	✔	✔				7
Dehydroepiandrosterone sulfate	7	HMDB0001032	✔	✔			✔	✔							4
Testosterone	9	HMDB0000234		✔		✔	✔	✔	✔	✔	✔			✔	8
Estrone	7	HMDB0000145		✔	✔		✔	✔				✔		✔	6
2-methoxyestrone	6	HMDB0000010		✔	✔		✔					✔			4
Pregnanetriol	6	HMDB0006070	✔	✔			✔					✔			4
Pregnanediol	6	HMDB0004025	✔									✔			2
Androsterone	6	HMDB0000031	✔	✔	✔		✔							✔	5
2-hydroxyestrone	6	HMDB0000343		✔	✔		✔					✔			4
Estriol	7	HMDB0000153			✔		✔					✔			3
16-epiestriol	6	NA			✔		✔					✔			3
16α-hydroxyestrone	6	HMDB0000335			✔		✔					✔			3
Etiocholanolone	6	HMDB0000490	✔		✔		✔								3
Androstenedione	5	HMDB0000053	✔	✔			✔								3
Dihydrotestosterone	5	HMDB0002961	✔	✔			✔							✔	4
16-ketoestradiol	5	HMDB0000406			✔		✔					✔			3
4-hydroxyestrone	5	HMDB0005895			✔		✔					✔			3

17-epiestriol	5	HMDB0000356			✔		✔								2
Cortisone	5	HMDB0002802	✔	✔		✔			✔	✔	✔				6
Tetrahydrodeoxycortisol	4	HMDB0005972	✔												1
Progesterone	4	HMDB0001830	✔	✔		✔			✔	✔	✔				6
Androstenediol	4	HMDB0003818					✔							✔	2
2-hydroxyestrone-3-methyl ether	4	NA			✔		✔								2
4-methoxyestrone	4	HMDB0060088		✔			✔					✔			3
Epitestosterone	3	HMDB0000628		✔		✔		✔	✔	✔	✔				6
ADT- sulfate	3	HMDB0002759		✔	✔										2
Androstenetriol	3	HMDB0000550	✔									✔			2
Tetrahydrocortisol	3	HMDB0000949	✔				✔								2
Tetrahydrocortisone	3	HMDB0000903	✔				✔								2
Estrone-sulfate	3	HMDB0001425		✔			✔								2
β-cortolone	3	HMDB0013221	✔				✔								2
α-cortolone	3	HMDB0003128	✔				✔								2
α-cortol	3	HMDB0003180	✔				✔								2
Corticosterone	3	HMDB0001547	✔	✔		✔			✔	✔					5
Tetrahydrocorticosterone	3	HMDB0000268	✔				✔					✔			3
4-androsten-3β,17β-diol disulfate 2	3	NA		✔	✔										2
androstane-3α, 17β-diol 17-glucuronide	3	NA		✔			✔								2
17α-hydroxypregnenolone	3	HMDB0000363	✔												1
ADT-glucuronide	3	HMDB0002829					✔								1

4-Methoxyestradiol	3	HMDB0012782					✓					✓			2
Allo-tetrahydrocortisol	2	HMDB0000526	✓					✓				✓			3
2-hydroxyestradiol	2	HMDB0000338			✓		✓								2
Tetrahydro-11-dehydrocorticosterone	2	NA					✓					✓			2
11-deoxycorticosterone	2	HMDB0000016	✓												1
6β-hydroxycortisol	2	NA	✓												1
17-hydroxyprogesterone	2	HMDB0000374	✓												1
11-deoxycortisol	2	HMDB0000015	✓												1
Epiandrosterone sulfate	2	HMDB0062657		✓	✓										2
Pregnenolone sulphate	2	HMDB0000774		✓											1
5α-androstan-3β,17β-diol disulfate	2	NA		✓											2
Pregnen-diol disulfate	2	NA		✓	✓										2
Cholest-5-en-3-ol	2	NA											✓	✓	2
Cholestane-tetrol glucuronide	2	NA		✓				✓							2
18-hydroxycorticosterone	1	HMDB0000319	✓												1
Cortolone-3-glucuronide	1	HMDB0010320		✓											1
Allo-tetrahydro-corticosterone	1	HMDB0000449					✓								1
Androstanediol	1	HMDB0000495						✓							1
Cholesterol sulfate	1	HMDB0000653						✓							1
β-sitosterol	1	HMDB0000852											✓		1
Lanosterol	1	HMDB0001251											✓		1
Epi-pregnanolone	1	HMDB0001471	✓												1

Dihydrocortisol	1	HMDB0003259	✓						1
2-methoxy-estrone-3-glucuronide	1	HMDB0004482					✓		1
Estrone glucuronide	1	HMDB0004483					✓		1
Tetrahydro-11- deoxycortisol	1	HMDB0005972	✓						1
5α-dihydrotestosterone sulfate	1	HMDB0006278		✓					1
Dihydrocortisone	1	HMDB0006758	✓						1
2-methoxy-estradiol- 3-glucuronide	1	HMDB0006765					✓		1
Cholesterol glucuronide	1	HMDB0010330						✓	1
Hydroxyandrosterone-3-glucuronide	1	HMDB0010351						✓	1
Tetrahydroaldosterone-3-glucuronide	1	HMDB0010357						✓	1
7α-hydroxy-3-oxo-4-cholestenoate	1	HMDB0012458		✓					1
5α-pregnan-3β, 20α-diol disulfate	1	HMDB0094650		✓					1
16α-hydroxy DHEA 3-sulfate	1	HMDB0062544			✓				1
11β-hydroxy-etiocholanolone	1	NA					✓		1
6β-hydroxycortisol	1	NA	✓						1
11-oxo-pregnanetriol	1	NA	✓						1
16-hydroxy-DHEA	1	NA	✓						1
5-pregnenediol	1	NA	✓						1
5-pregnenetriol	1	NA	✓						1
5α-tetrahydro-11-dehydrocorticosterone	1	NA	✓						1
Androstendiol-17β	1	NA	✓						1
5-pregnen,3α,16α,20α-triol	1	NA	✓						1

5-pregnen,3α,17α,20α-triol	1	NA	✔												1
6-hydroxy-pregnanolone	1	NA	✔												1
Hexahydro-11-deoxycortisol	1	NA	✔												1
21-deoxy-tetrahydrocortisol	1	NA	✔												1
Hexahydrocorticosterone	1	NA	✔												1
4-OHE1-1-N7Gua	1	NA		✔											1
4-OHE1-1-N3Ade	1	NA		✔											1
2-OHE1-6-N3Ade	1	NA		✔											1
Androstane-3α, 17β-diol 3-glucuronide	1	NA					✔								1
Androsten-3β -17β -diol	1	NA			✔										1
Androstane-3β -17β -diol	1	NA		✔											1
3β,16α-dihydroxyandrostenone sulfate	1	NA		✔											1
Pregnanetriol glucuronide	1	NA		✔											1
4-androsten-3α,17α-diol monosulfate	1	NA		✔											1
Cholesta-3,5-diene	1	NA												✔	1
4-Androsten-3β,17β-diol disulfate	1	NA		✔											1
5α-androstan-3α,17α diol monosulfate	1	NA		✔											1
Steroid monosulfate 1	1	NA		✔											1
4-androsten- 3β,17β -diol disulfate 1	1	NA		✔											1
Andro steroid monosulfate 1	1	NA		✔											1

PC: Prostate cancer; AC: Adrenal cancer; BC: Breast cancer; BlC: Bladder cancer; EC: Endometrial cancer; LC: Liver cancer; KC: Kidney cancer; TC: Testicle cancer; NET: Neuroendocrine Tumor; OC: Ovarian cancer; OrC: Oral cancer; E/GC: Esophagus/Gastric cancer

Table S5. The characteristics of significantly enriched pathways analysis.

	Pathway name	Match status	Included steroids	p	-log (p)	FDR	Impact
**Pathway enrichment**	Steroidogenesis	10/42	Corticosterone, tetrahydrocorticosterone, cortisol, deoxycorticosterone, progesterone, 17-hydroxyprogesterone, cortexolone, 17a-hydroxypregnenolone, tetrahydrocortisone, tetrahydrocortisol	3.65E-06	12.52	3.62E-04	0.074162
	Androgen and Estrogen Metabolism	6/29	Estrone sulfate, androstenedione, 17-hydroxyprogesterone, 17a-hydroxypregnenolone, dehydroepiandrosterone sulfate, dihydrotestosteron	9.57E-04	6.952	0.047357	0.13184
	Androstenedione Metabolism*	5/23	Androstenedione, androsterone, estrone sulfate, etiocholanolone	0.0021096	6.1613	0.069617	0.16016

*Only four steroids were shown in the returning results

PRISMA 2009 Checklist

Table S6. PRISMA checklist.

Section/topic	#	Checklist item	Reported on page #
**TITLE**			
Title	1	Identify the report as a systematic review, meta-analysis, or both.	1
**ABSTRACT**			
Structured summary	2	Provide a structured summary including, as applicable: background; objectives; data sources; study eligibility criteria, participants, and interventions; study appraisal and synthesis methods; results; limitations; conclusions and implications of key findings; systematic review registration number.	1
**INTRODUCTION**			
Rationale	3	Describe the rationale for the review in the context of what is already known.	2
Objectives	4	Provide an explicit statement of questions being addressed with reference to participants, interventions, comparisons, outcomes, and study design (PICOS).	2
**METHODS**			
Protocol and registration	5	Indicate if a review protocol exists, if and where it can be accessed (e.g., Web address), and, if available, provide registration information including registration number.	NA
Eligibility criteria	6	Specify study characteristics (e.g., PICOS, length of follow-up) and report characteristics (e.g., years considered, language, publication status) used as criteria for eligibility, giving rationale.	20
Information sources	7	Describe all information sources (e.g., databases with dates of coverage, contact with study authors to identify additional studies) in the search and date last searched.	20
Search	8	Present full electronic search strategy for at least one database, including any limits used, such that it could be repeated.	20
Study selection	9	State the process for selecting studies (i.e., screening, eligibility, included in systematic review, and, if applicable, included in the meta-analysis).	20
Data collection process	10	Describe method of data extraction from reports (e.g., piloted forms, independently, in duplicate) and any processes for obtaining and confirming data from investigators.	20-21
Data items	11	List and define all variables for which data were sought (e.g., PICOS, funding sources) and any assumptions and simplifications made.	20-21
Risk of bias in individual studies	12	Describe methods used for assessing risk of bias of individual studies (including specification of whether this was done at the study or outcome level), and how this information is to be used in any data synthesis.	NA
Summary measures	13	State the principal summary measures (e.g., risk ratio, difference in means).	NA
Synthesis of results	14	Describe the methods of handling data and combining results of studies, if done, including measures of consistency (e.g., I^2) for each meta-analysis.	NA

PRISMA 2009 Checklist

Section/topic	#	Checklist item	Reported on page #
Risk of bias across studies	15	Specify any assessment of risk of bias that may affect the cumulative evidence (e.g., publication bias, selective reporting within studies).	NA
Additional analyses	16	Describe methods of additional analyses (e.g., sensitivity or subgroup analyses, meta-regression), if done, indicating which were pre-specified.	21
**RESULTS**			
Study selection	17	Give numbers of studies screened, assessed for eligibility, and included in the review, with reasons for exclusions at each stage, ideally with a flow diagram.	2-3
Study characteristics	18	For each study, present characteristics for which data were extracted (e.g., study size, PICOS, follow-up period) and provide the citations.	3-10
Risk of bias within studies	19	Present data on risk of bias of each study and, if available, any outcome level assessment (see item 12).	NA
Results of individual studies	20	For all outcomes considered (benefits or harms), present, for each study: (a) simple summary data for each intervention group (b) effect estimates and confidence intervals, ideally with a forest plot.	11-13
Synthesis of results	21	Present results of each meta-analysis done, including confidence intervals and measures of consistency.	NA
Risk of bias across studies	22	Present results of any assessment of risk of bias across studies (see Item 15).	12
Additional analysis	23	Give results of additional analyses, if done (e.g., sensitivity or subgroup analyses, meta-regression [see Item 16]).	12
**DISCUSSION**			
Summary of evidence	24	Summarize the main findings including the strength of evidence for each main outcome; consider their relevance to key groups (e.g., healthcare providers, users, and policy makers).	16-20
Limitations	25	Discuss limitations at study and outcome level (e.g., risk of bias), and at review-level (e.g., incomplete retrieval of identified research, reporting bias).	16-20
Conclusions	26	Provide a general interpretation of the results in the context of other evidence, and implications for future research.	21
**FUNDING**			
Funding	27	Describe sources of funding for the systematic review and other support (e.g., supply of data); role of funders for the systematic review.	21

From: Moher D, Liberati A, Tetzlaff J, Altman DG, The PRISMA Group (2009). Preferred Reporting Items for Systematic Reviews and Meta-Analyses: The PRISMA Statement. PLoS Med 6(7): e1000097. doi:10.1371/journal.pmed1000097

For more information, visit: **www.prisma-statement.org**.

Figure S1. Descriptive summary of the included studies. (a) The number of the included studies each year, (b) Type of cancer, (c) Study purpose

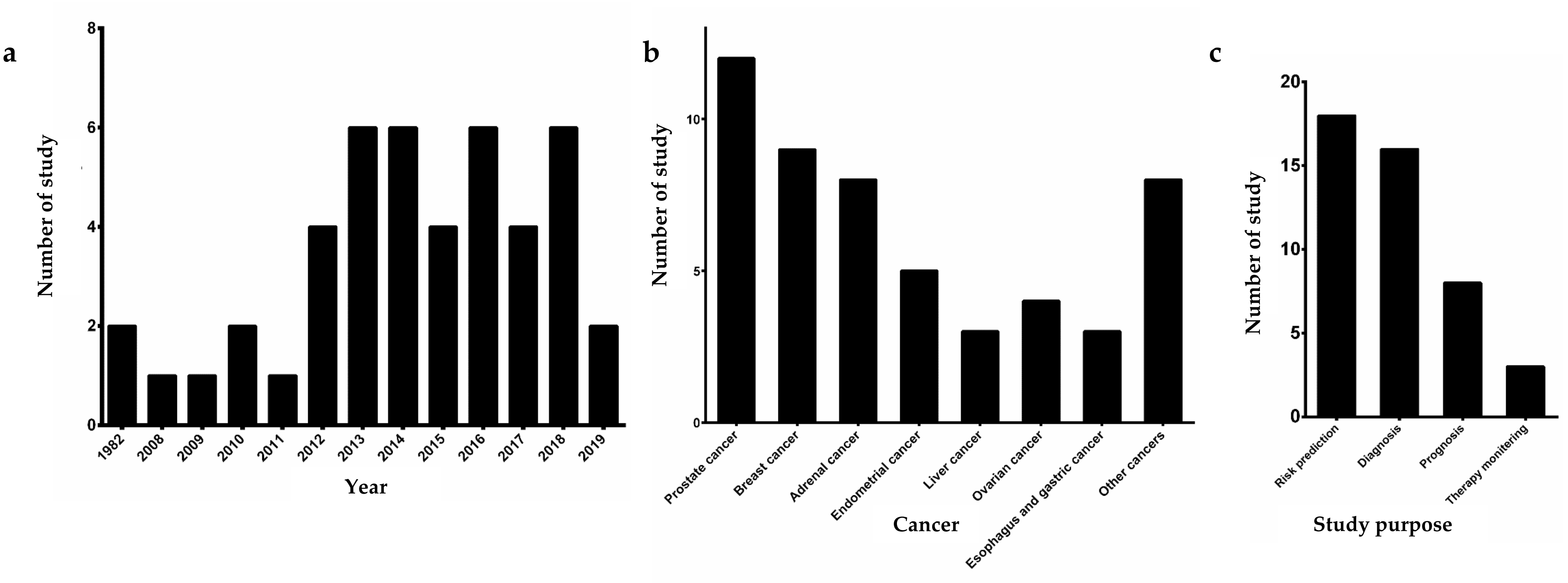

Figure S2. Pathway visualization of the two steroid pathways altered in cancers. (a) Androgen and Estrogen Metabolism, (b) Androstenedione Metabolism. The red boxes refer to the potentially altered steroids in the included studies.

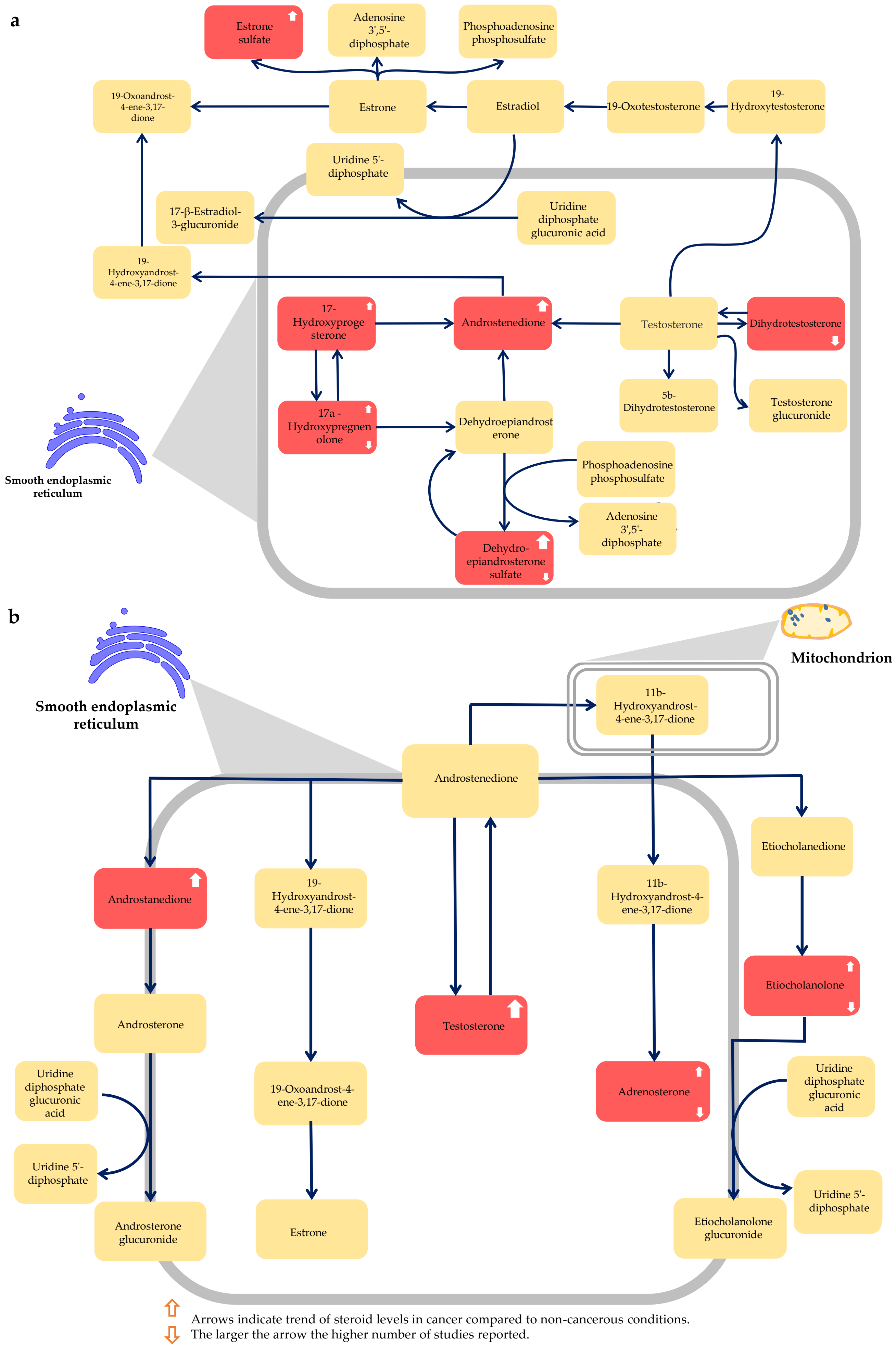

Supplement: Supplementary file 1 [file metabolites-09-00199-s001.pdf]
